# *PAQR6* as a prognostic biomarker and potential therapeutic target in kidney renal clear cell carcinoma

**DOI:** 10.3389/fimmu.2024.1521629

**Published:** 2024-12-17

**Authors:** Tao Zou, Zongming Jia, Jixiang Wu, Xuxu Liu, Minghao Deng, Xuefeng Zhang, Yuxin Lin, Jigen Ping

**Affiliations:** ^1^ Department of Urology, The First Affiliated Hospital of Soochow University, Suzhou, China; ^2^ Department of Neurology Children’s Hospital of Chongqing Medical University, National Clinical Research Center for Child Health and Disorders, Ministry of Education Key Laboratory of Child Development and Disorders, Chongqing, China; ^3^ Department of Urology, Nantong Hospital of Traditional Chinese Medicine, Nantong, China; ^4^ Center for Systems Biology, Soochow University, Suzhou, China

**Keywords:** kidney renal clear cell carcinoma, immune infiltration, prognostic biomarker, PAQR6, angiogenesis

## Abstract

**Background:**

Progestin And AdipoQ Receptor Family Member VI (*PAQR6*) plays a significant role in the non-genomic effects of rapid steroid responses and is abnormally expressed in various tumors. However, its biological function in kidney renal clear cell carcinoma (KIRC) and its potential as a therapeutic target remain underexplored.

**Methods:**

In this study, *PAQR6* was identified as a critical oncogene by WGCNA algorithm and differential gene expression analysis using TCGA - KIRC and GSE15641 data. The differences in *PAQR6* expression and its association with KIRC survival outcomes were investigated, and transcriptomic data were used to further elucidate *PAQR6*’s biological functions. Moreover, XCELL and single - cell analysis assessed the correlation between *PAQR6* expression and immune infiltration. TIDE algorithm was used to assess how well various patient cohorts responded to immune checkpoint therapy. Finally, the role of *PAQR6* in the development of KIRC was verified through EdU, scratch assays, and Transwell assays.

**Results:**

Our findings suggest that elevated expression of *PAQR6* is linked to a poor prognosis for KIRC patients. Functional enrichment analysis demonstrated that *PAQR6* is primarily involved in angiogenesis and pluripotent stem cell differentiation, which are crucial in mediating the development of KIRC. Additionally, we established a ceRNA network that is directly related to overall prognosis, further supporting the role of *PAQR6* as a prognostic biomarker for KIRC.

**Conclusion:**

Using both computational and experimental methods, this study leads the charge in discovering and verifying *PAQR6* as a prognostic biomarker and possible therapeutic target for KIRC. In the future, to determine its molecular mechanism in KIRC carcinogenesis, more *in vivo* research will be carried out.

## 1 Introduction

Kidney Renal Clear Cell Carcinoma (KIRC), the most prevalent subtype of renal cell carcinoma (RCC), accounts for approximately 70–80% of RCC cases and is highly aggressive with frequent metastasis and recurrence (1). Current treatments, including laparoscopic partial nephrectomy and radical nephrectomy, are effective for localized tumors but have limited impact on advanced KIRC (2, 3). For advanced cases, systemic medication therapies, such as tyrosine kinase inhibitors (TKIs) and immune checkpoint inhibitors (ICIs), offer some benefit (4, 5). ICIs, by reducing the suppression of immune responses, with the tumor immune response involved, by interfering with the interactions between PD-1 and PD-L1/PD-L2 or the binding of CTLA-4 to CD80/CD86. However, ICIs show variable response rates, with a lack of reliable biomarkers to predict therapeutic outcomes (6–9). The tumor microenvironment, particularly immune checkpoint regulation and T cell dysfunction, plays a critical role in KIRC progression (10–12). Despite advances, novel biomarkers and therapeutic targets are urgently needed to improve outcomes, especially for advanced cases. Exploring oncogenic pathways and immune mechanisms offers opportunities to better understand KIRC pathophysiology and guide precision therapies.

Among the potential therapeutic targets for KIRC, the *PAQR* family in the human genome comprises 11 members (*PAQR1* to *PAQR11*), has emerged as a significant player in metabolism and carcinogenesis (13, 14). The *PAQR1-4* subgroup includes adiponectin-related receptors, with AdipoR1 (*PAQR1*) and AdipoR2 (*PAQR2*) playing key roles in fatty acid oxidation and glucose uptake (15). *PAQR3* has been linked to cell cycle regulation, particularly in tumor cell proliferation and apoptosis (16). The *PAQR5-9* subgroup consists of membrane progesterone receptors (mPRs), including *PAQR5* (mPRγ), *PAQR6* (mPRδ), *PAQR7* (mPRα), *PAQR8* (mPRβ), and *PAQR9* (mPRϵ), which are involved in cell cycle processes and malignant biological behaviors of tumors (14, 17). For instance, *PAQR5* and *PAQR8* are differentially expressed in ovarian cystadenomas, borderline tumors, and carcinomas, and are also considered potential prognostic biomarkers for endometrial cancer (17, 18). Additionally, *PAQR7* has been proven to stimulate cell proliferation and motility in human glioblastoma cells, implicating it in glioblastoma progression (19). Notably, research by Li Zhou et al. demonstrated that progesterone inhibits the growth and metastasis of triple-negative breast cancer through *PAQR7* (20). Notably, PAQR6 has been shown to modulate the MAPK signaling pathway and promote prostate cancer progression (21). However, its role in KIRC remains largely unexplored, presenting an opportunity to uncover its potential as both a prognostic biomarker and a therapeutic target.

The expression of *PAQR6* in KIRC and its prognostic importance were examined in this work using extensive computational and experimental investigations. Additionally, we used gene enrichment analysis to investigate *PAQR6*’s possible involvement in KIRC. According to our findings, *PAQR6* regulates the angiogenesis and pluripotent stem cell development pathways in KIRC and interacts with the well-known oncogene EZH2 (22, 23). Additionally, we created a ceRNA network that includes *PAQR6*, and immune-related analyses suggest that *PAQR6* might act as a potential target for immunotherapy of KIRC. *In vitro* EdU assays, scratch assays, and Transwell assays corroborated our computational findings, providing further evidence that *PAQR6* is a novel biomarker for KIRC management.

## 2 Materials and methods

### 2.1 Data sets and patient samples

In this study, differential analysis was conducted utilizing the mRNA sequencing data from the TCGA-KIRC dataset, which includes 72 normal samples and 532 KIRC samples (**Supplementary Table S1**), and 32 KIRC and 23 normal samples from the GSE15641 dataset (**Supplementary Table S2**) in the Gene Expression Omnibus (GEO) database. The clinical information of 39 KIRC samples in the GSE29609 (**Supplementary Table S3**) dataset was used for WGCNA analysis. From January 2024 to April 2024, 20 pairs of cancerous and nearby normal tissues from KIRC patients were surgically removed at Soochow University’s First Affiliated Hospital. Postoperative pathology verified the specimens. Prior to the procedure, anti-tumor treatment was not administered to any of the patients. The Ethics Committee of Soochow University’s First Affiliated Hospital granted approval for this study under the number 2024-395. Written informed consent forms were signed by each patient.

### 2.2 Differential expression analysis and WGCNA

The investigation of mRNA differential expression was conducted using the R software’s Limma package (version 3.40.2). Differential analysis was carried out using the GSE15641 and TCGA-KIRC datasets, and the screening criteria were Log2 (Fold Change) > 2 or Log2 (Fold Change) < -2 and *P* < 0.05 (**Supplementary Figures S1A, B**). In this study, all selected genes were upregulated. The GSE29609 dataset, which includes prognostic information for 39 cases of KIRC, was analyzed to identify the module with the highest correlation using WGCNA. To meet the assumption of a scale-free network as closely as possible, it was necessary to determine an appropriate value for the adjacency matrix weight parameter, power. The power value was set to range from 1 to 30, and the corresponding network correlation coefficients and mean connectivity were calculated. A higher correlation coefficient (with a maximum value of 1) indicates a closer fit to a scale-free network distribution. However, to ensure the robustness of the network, the power value was selected to balance a sufficiently high correlation coefficient with adequate gene connectivity. In this analysis, the power value was set to 7, as shown in **Supplementary Figures S1C, D**. Based on the selected power value, a weighted gene co-expression network model was constructed, resulting in the division of genes into six modules. The gray module represents a collection of genes that could not be assigned to any specific module (**Supplementary Figure S1E**). Among the modules, the brown module exhibited the highest correlation, with a correlation coefficient of 0.34 (**Supplementary Figure S1F**).

### 2.3 Gene enrichment analysis

The data were examined using functional enrichment to further validate the possible roles of the possible targets. A popular technique for annotating genes with functions is Gene Ontology (GO), particularly for molecular function (MF), biological process (BP), and cellular component (CC). Gene functions and related advanced genomic functional information can be analyzed with the use of the useful Kyoto Encyclopedia of Genes and Genomes (KEGG) enrichment analysis. The ClusterProfiler package in R was used for GO and KEGG enrichment analysis, as well as to investigate possible aspects of Gene Set Enrichment Analysis (GSEA), in order to better understand the target gene’s carcinogenic role (24). To control for false positives resulting from multiple testing, we applied the Benjamini-Hochberg method to adjust the p-values, calculating the false discovery rate (FDR). Enriched terms with an adjusted p-value (FDR) of less than 0.05 were considered statistically significant.

### 2.4 Immune infiltration analysis

In order to guarantee a trustworthy assessment of the immune score outcomes, we utilized the R software package immunedeconv. Every algorithm had distinct benefits and had undergone extensive testing. The XCELL approach was used for this investigation since it evaluates a greater variety of immune cells. Additionally, we identified immune cells with prognostic value using the LASSO algorithm. R Foundation for Statistical Computing version 4.0.3 was used to implement all of the previously discussed analytic techniques and R packages.

### 2.5 CeRNA network analysis

The ENCORI and TarBase v.8 databases were used to analyze *PAQR6*-related miRNA. The ENCORI database was used to analyze circRNA related to miRNA (25, 26).

### 2.6 Cell cultures and viral infection

769P cell was cultured in RPMI 1640 medium (cytiva, UK) containing 10% fetal bovine serum (Gibco, USA) and the cell was cultured in a 37°C cell incubator containing 5% CO2. *PAQR6* knockdown lentivirus were purchased from Shanghai Genechem (China). Viral infection was performed according to the instructions, and then screening was performed with puromycin.

### 2.7 Western blot

RIPA lysate (Beyotime, China) combined with a proteinase inhibitor was used to extract the proteins. After loading proteins onto an SDS-PAGE gel, they were moved onto a PVDF membrane. After blocking the membrane with 5% skim milk for two hours at room temperature, the matching primary antibody (abs143408, absin) was incubated at 4°C for the entire night. Lastly, an enhanced chemiluminescence (ECL) kit (Beyotime) was used to expose the membrane after it had been treated with secondary antibodies coupled with horseradish peroxidase.

### 2.8 EdU assay

The supplier of the EdU assay kit was Beyotime Company (C0078S, China). Transfected cells were exposed to EdU reagent for two hours in accordance with the manufacturer’s instructions. The cells were then incubated with 0.3% Triton X-100 for 15 minutes at room temperature after being fixed with 4% paraformaldehyde for 15 minutes. Lastly, cells were stained using Hoechst and fluorescent dye. The pictures were taken with a Nikon TI2-D-PD inverted microscope (Japan).

### 2.9 Scratch assay

Following the creation of stably transfected cell lines, the cell plate was scratched with 200 μl of Eppen-dorf Tip, and the cells were then rinsed two or three times with PBS. 1% fetal bovine serum is still used to cultivate cells (Gibco, USA). Use an inverted microscope to see how the cells in each plate change at 0 and 12 hours.

### 2.10 Transwell assay

A coating of Matrigel matrix glue (Corning, USA) (matrix glue: serum-free medium=1:6) was applied to the upper chamber. Cells are resuspended in serum-free media 36 hours after transfection. Next, 500 μl of complete media was added to the lower chamber, and 5×104 cells were transferred to the upper chamber. Fix the cells in the upper chamber with 4% paraformaldehyde for 30 minutes at room temperature after 24 hours, and then stain them for 20 minutes with crystal violet. The pictures were taken with a Nikon TI2-D-PD inverted microscope (Japan).

### 2.11 Statistical analysis

The expression of *PAQR6* in KIRC and normal kidney tissues was detected by the Wilcoxon rank-sum test. The log-rank test was used for prognostic analysis. It was deemed statistically significant when the *P* value was less than 0.05.

## 3 Results

### 3.1 *PAQR6* is highly expressed in KIRC

**Figure 1:**
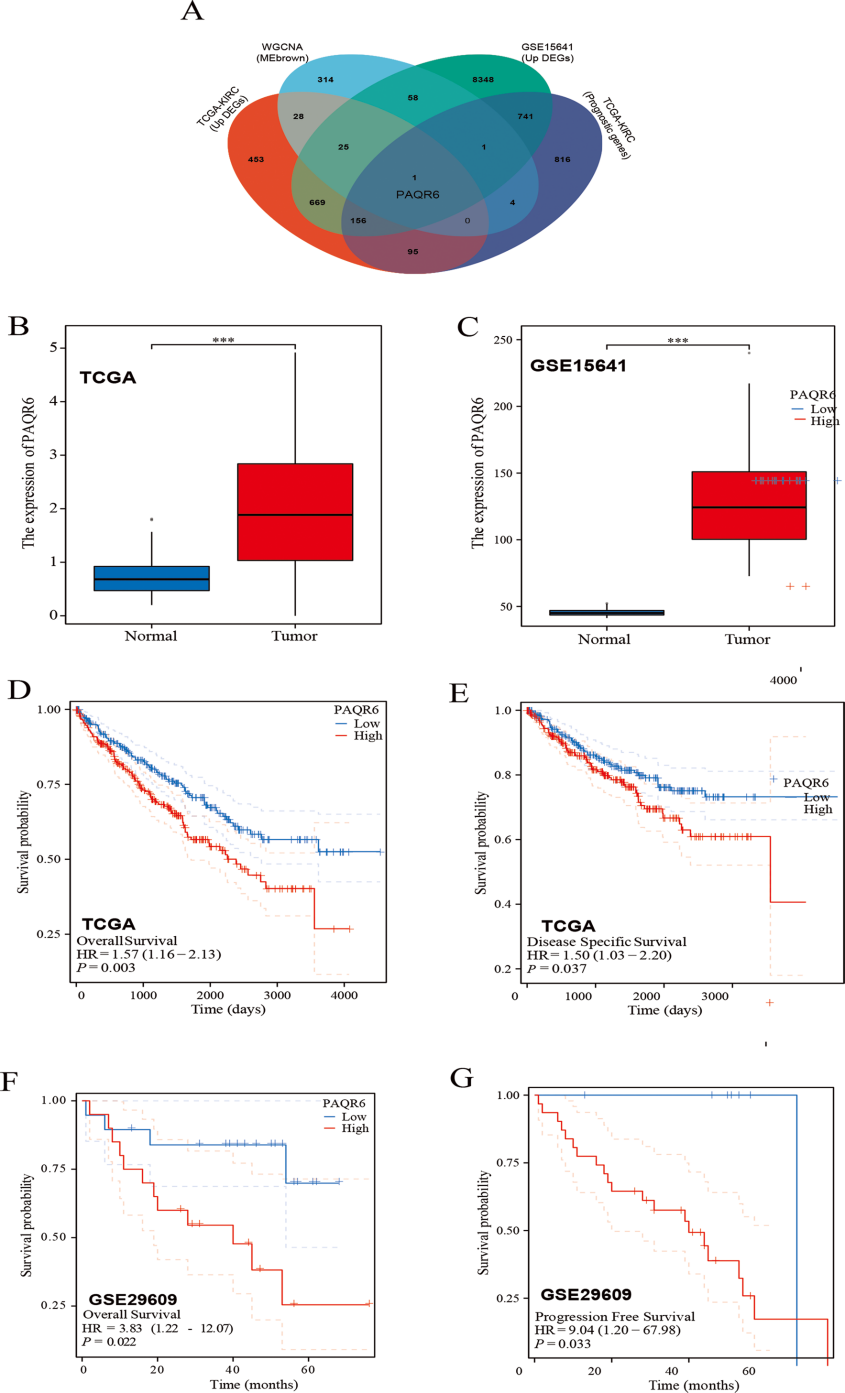
Patients with high expression of *PAQR6* have a poor prognosis. **(A)** Venn diagram identifying *PAQR6* as a key gene. **(B, C)** Comparison of *PAQR6* expression in KIRC and normal tissues in the TCGA-KIRC and GSE15641 databases. **(D)** Kaplan-Meier survival curve of OS for *PAQR6* expression in TCGA-KIRC dataset. **(E)** Kaplan-Meier survival curve of DSS for *PAQR6* expression in TCGA-KIRC dataset. **(F)** Kaplan-Meier survival curve of OS for *PAQR6* expression in the GSE29609 dataset. **(G)** Kaplan-Meier survival curve of PFS for *PAQR6* expression in the GSE29609 dataset. ***: P<0.001.

Two different datasets (TCGA-KIRC and GSE15641) were used to group by cancer and adjacent cancer tissues and then screened differentially expressed genes through differential analysis (**Supplementary Figures S1A, B**). Subsequently, based on WGCNA, we further screened the key genes related to the prognosis of KIRC (**Supplementary Figures S1C–F**). A Venn diagram was used to intersect the results from these analyses, leading to the identification of *PAQR6* as the key gene for subsequent investigations (**Figure 1A**). According to our findings, *PAQR6* was consistently upregulated in KIRC samples as compared to normal renal tissues (**Figures 1B, C**).

To assess the prognostic implications of *PAQR6*, we analyzed data from the TCGA-KIRC dataset and observed that high *PAQR6* expression was linked to lower rates of overall survival(OS) (**Figure 1D**). The finding was corroborated using the GSE29609 dataset (**Figure 1F**). To deepen our understanding, we performed disease-specific survival (DSS) and progression-free survival (PFS) analyses. DSS, which measures survival probability without death specifically attributable to KIRC, was analyzed using the TCGA-KIRC dataset (**Figure 1E**), providing insights into the relationship between *PAQR6* expression and cancer-specific mortality. Similarly, PFS, assessed using the GSE29609 dataset (**Figure 1G**), evaluates the time to disease progression, including tumor recurrence or metastasis, offering critical insights into whether elevated *PAQR6* expression is linked to more aggressive disease behavior or shorter recurrence-free intervals.

By combining DSS and PFS analyses, we provided a comprehensive evaluation of *PAQR6*’s prognostic significance, demonstrating its association with higher cancer-specific mortality and accelerated disease progression. In conclusion, our findings suggest that elevated *PAQR6* expression in KIRC is strongly linked to poor patient prognosis, underscoring its potential as a prognostic biomarker.

### 3.2 Differential analysis based on *PAQR6*

**Figure 2:**
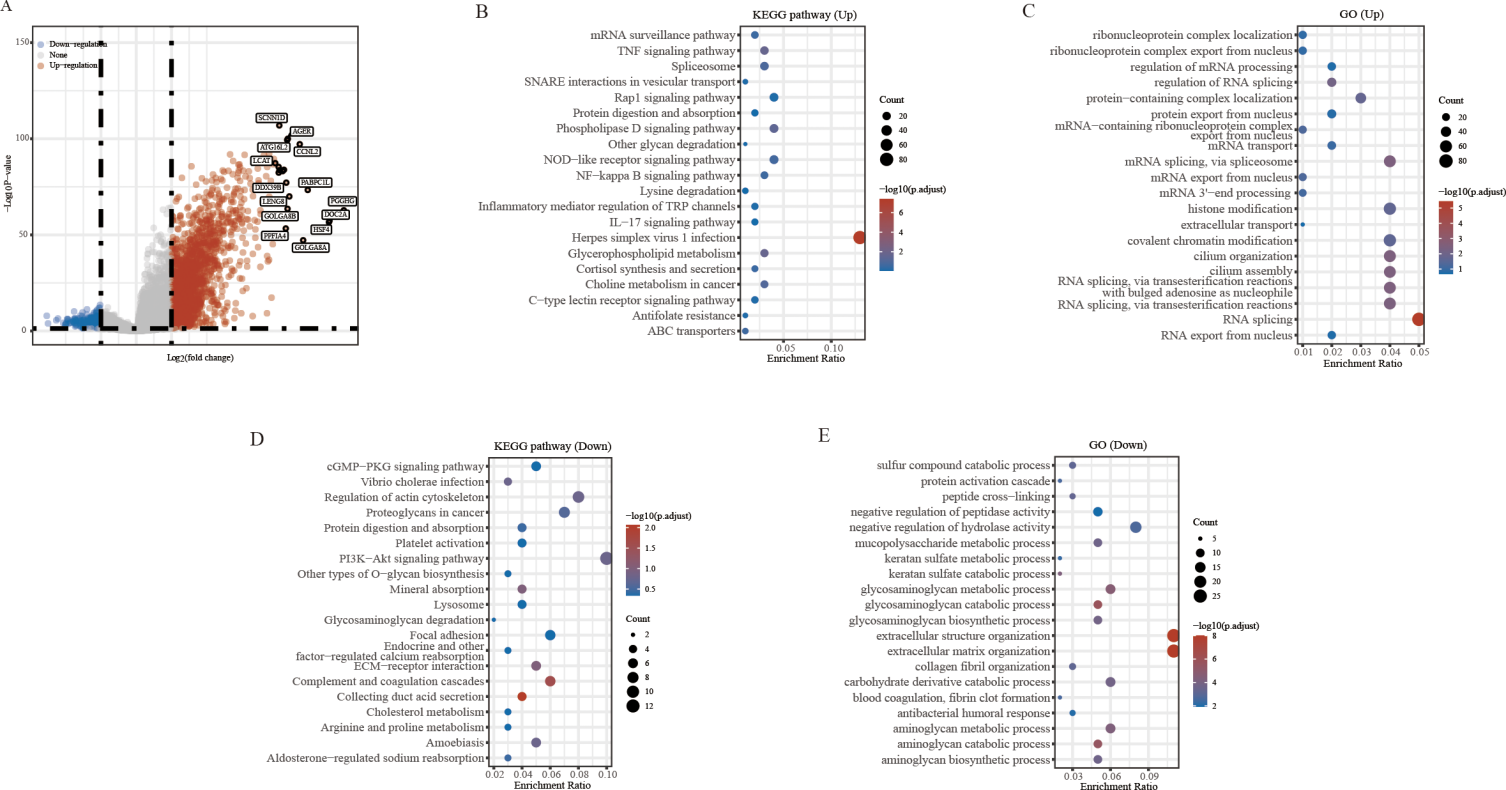
Differential analysis and enrichment analysis based on *PAQR6*. **(A)** Volcano plot of differential analysis of *PAQR6*. **(B, C)** KEGG and GO analysis of *PAQR6*-related upregulated genes. **(D, E)** KEGG and GO analysis of *PAQR6*-related downregulated genes.

In this study, we investigated the potential role of *PAQR6* in KIRC. To identify the differentially expressed genes related to *PAQR6*, we applied screening criteria of *P* < 0.05, Log2 (Fold Change) > 2 or Log2 (Fold Change) < -2 (**Figure 2A**). The function of *PAQR6* in KIRC was then further confirmed by functional enrichment analysis. KEGG pathway analysis revealed that upregulated genes were significantly enriched in pathways such as herpes simplex virus type 1 infection, Rap l signaling, phospholipase D signaling, and NOD-like receptor signaling (**Figure 2B**). These increased genes were implicated in RNA splicing, according to GO analysis, histone modification, covalent chromatin modification, and ciliary organization (**Figure 2C**). Conversely, KEGG analysis of downregulated genes identified their involvement in pathways like PI3K-Akt signaling, actin cytoskeleton regulation, cancer-related proteoglycans, and complement and coagulation cascades (**Figure 2D**). GO analysis revealed a connection between downregulated genes and extracellular matrix and structural organization, negative regulation of hydrolase activity, and glycosaminoglycan metabolic processes (**Figure 2E**).These findings indicate that *PAQR6* plays a complex role in KIRC through multiple biological processes.

### 3.3 Gene set enrichment analysis of *PAQR6* in KIRC

**Figure 3:**
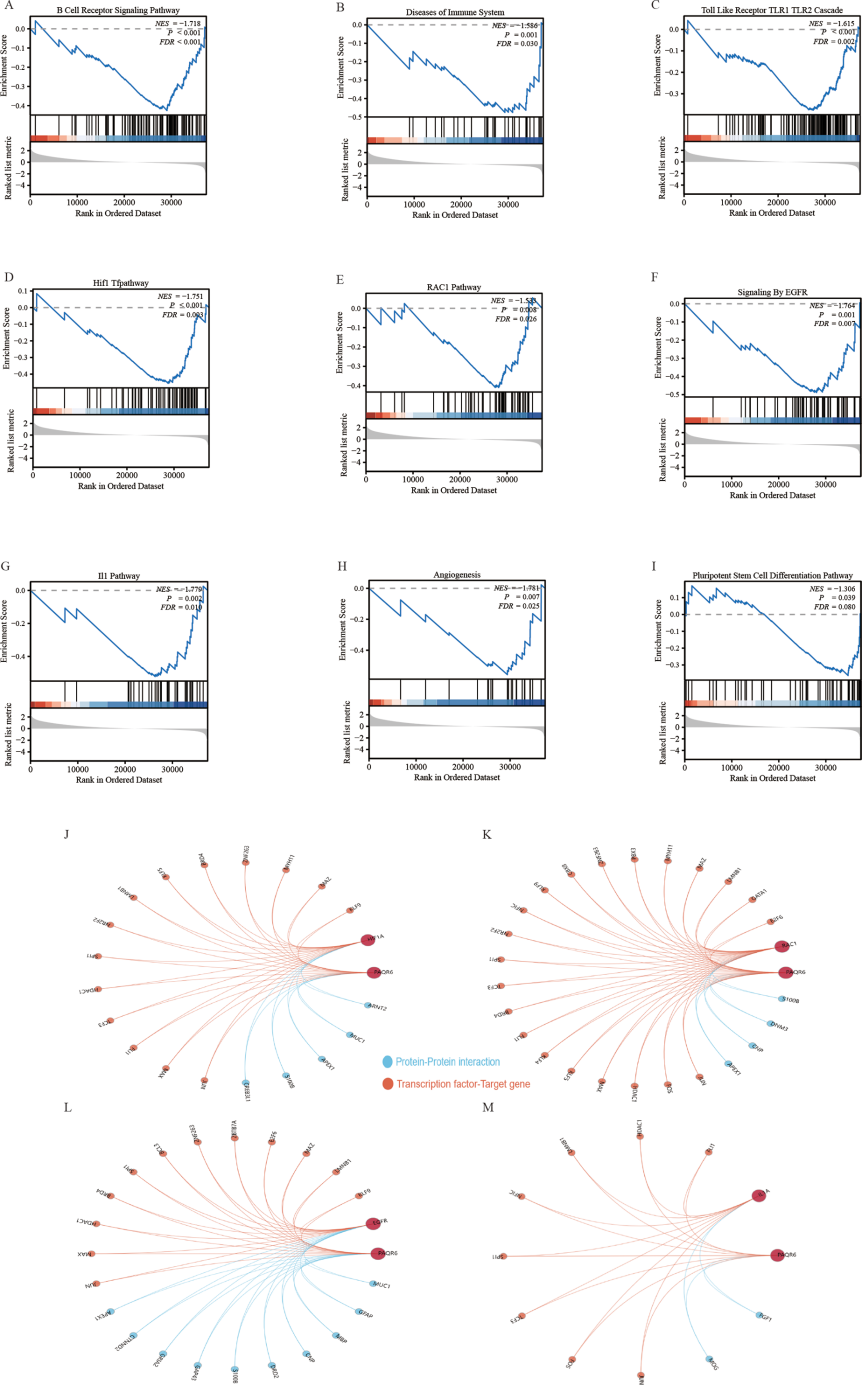
Gene set enrichment analysis based on *PAQR6*. **(A)** B cell receptor signaling pathway. **(B)** Immune system diseases. **(C)** Toll-like receptor TLR1 TLR2 cascade. **(D)** *HIF1* pathway. **(E)** *RAC1* pathway. **(F)** *EGFR* signaling. **(G)** *IL1* pathway. **(H)** Angiogenesis. **(I)** Pluripotent stem cell differentiation pathway. **(J–M)** Complementary genes and target genes shared by *PAQR6* with *HIF1A, RAC1, EGFR*, and *IL1A*.

General differential analysis methods such as GO and KEGG may overlook genes with subtle expression differences that are still biologically significant. These methods do not fully account for important factors like gene regulator networks and the functional significance of gene interactions. To overcome these limitations, we performed a more thorough analysis of *PAQR6* in KIRC using GSEA. Our findings revealed a significant correlation between *PAQR6* and the immune microenvironment pathway of KIRC, including B cell receptor signaling, Toll-like receptor 1 and Toll-like receptor 2 cascades (**Figures 3A–C**). GSEA also confirmed a significant association between *PAQR6* and pathways involved in angiogenesis and pluripotent stem cell differentiation (**Figures 3H, I**). Key target genes regulated by *PAQR6*, such as *HIF1A, RAC1, EGFR*, and *IL1A*, were identified (**Figures 3D–G**). Finally, the Gendoma database was used to identify the common interaction proteins between *PAQR6* and these four target genes (**Figures 3J–M**). Protein interaction analysis further supported these associations, linking *PAQR6* with angiogenesis and stem cell differentiation processes. In conclusion, the GSEA analysis provided detailed understanding of the multiple potential functions of *PAQR6* in KIRC.

### 3.4 Immune-related analysis of *PAQR6*

**Figure 4:**
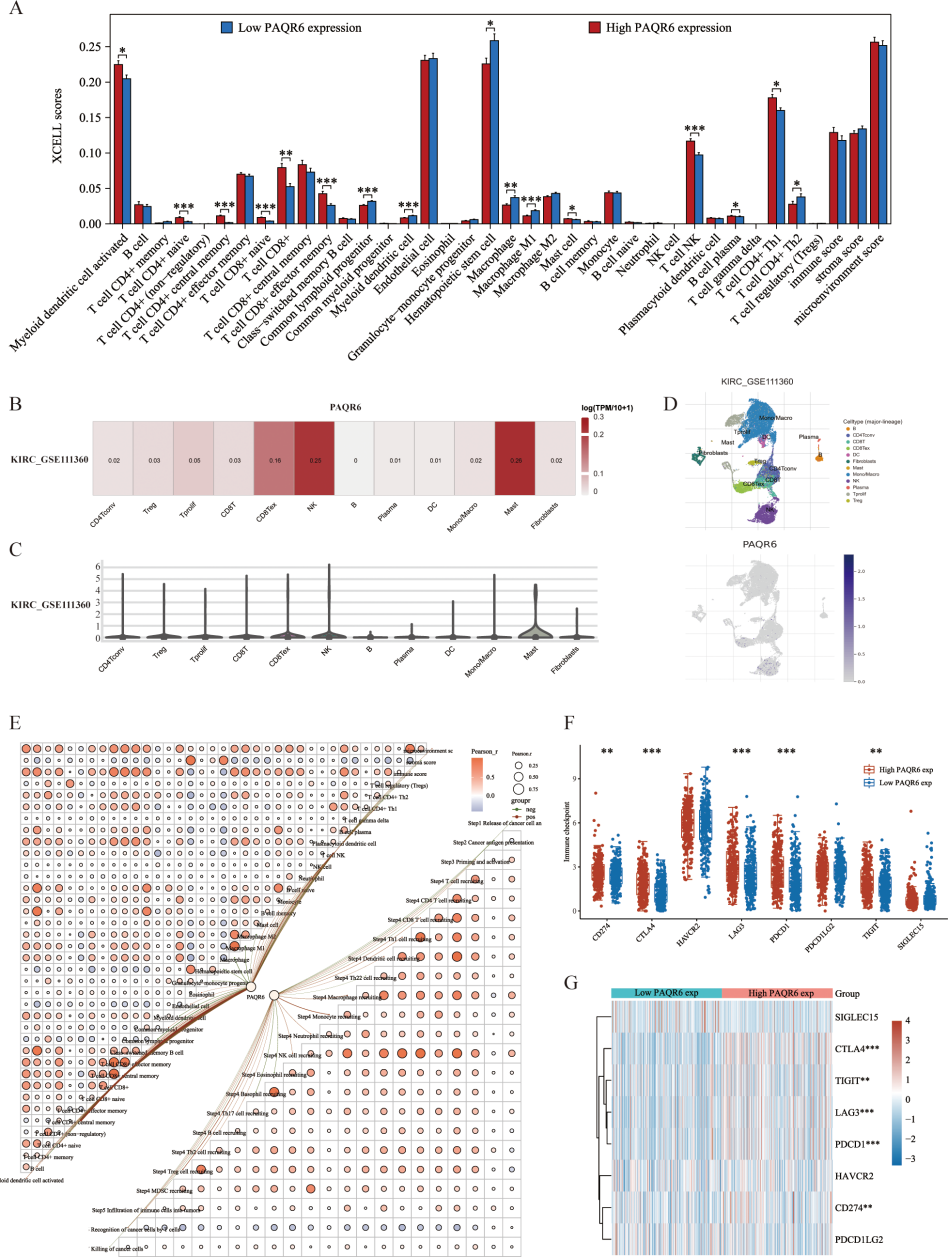
*PAQR6* is closely related to the immune microenvironment. **(A)** Correlation of *PAQR6* expression with immune cells. **(B–D)** Analysis of *PAQR6* and immune cell infiltration levels in KIRC in the GSE111360 dataset. **(E)** Correlation of *PAQR6* expression with immune scores and the correlation of immune scores themselves. **(F)** Correlation analysis of *PAQR6* expression and immune checkpoint genes. **(G)** Analysis of *PAQR6* expression and response to immune checkpoint inhibitor therapy. (*P < 0.05, **p < 0.01, ***P < 0.001).

In this study, 38 immune cell types from the TCGA-KIRC dataset were evaluated using the XCELL method, identifying 12 that were significantly associated with KIRC prognosis. 5 key immune cell types were incorporated into a prognostic model: hematopoietic stem cells, monocytes, naive B cells, NK T cells, and CD4+Th1 T cells (**Supplementary Figures S2A–L**, **S3A, B**). A risk score formula based on the expression of these cells demonstrated a significantly poorer prognosis for high-risk patients, who also exhibited reduced responses to immune checkpoint inhibitors, as indicated by elevated TIDE scores (**Supplementary Figures S3C–E**). Hematopoietic stem cells, monocytes, and naive B cells were all validated by Cox regression analysis as possible prognostic indicators for KIRC (**Supplementary Figures S3F, G**). Further investigation showed that the expression of various immune regulatory genes varied significantly between the high-risk and low-risk groups, highlighting the distinct immunological profiles of these groups (**Supplementary Figures S4A–C**). The infiltration scores of 16 immune cell types showed notable differences between the groups with high and low *PAQR6* expression, according to our analysis of the relationship between *PAQR6* expression and immune cell infiltration in KIRC (**Figure 4A**). These results highlight *PAQR6*’s crucial function in the immune microenvironment of KIRC, with significant correlations to both immune stimulatory and suppressive factors, as well as immune cell infiltration.

Single-cell analysis was used to investigate the connection between *PAQR6* expression and immune cell infiltration in KIRC. In the GSE111360 dataset (**Supplementary Table S4**), *PAQR6* expression was significantly correlated with various immune cell types, including CD4Tconv, Treg cells, Tprolif cells, CD8T cells, CD8Tex cells, NK cells, B cells, plasma cells, DC cells, monocytes/macrophages, mast cells, and fibroblasts (**Figures 4B, C**). A heatmap was generated to illustrate these correlations (**Figure 4D**). A correlation network graph was used to illustrate the substantial relationship between *PAQR6* expression and immunological scores that was found through additional research utilizing the XCELL and TIP algorithms (**Figure 4E**).

We investigated the regulatory effects of *PAQR6* on CD8+ T cells, which have been shown to have a prognostic significance. Our findings indicate that *PAQR6* may affect KIRC prognosis via modifying CD8+ T cells. Additionally, we examined the relationship between *PAQR6* expression and immune checkpoint genes, revealing notable distinctions between groups with high and low *PAQR6* expression (**Figure 4F**). According to TIDE study, a poor prognosis after immune checkpoint inhibitor therapy is linked to increased *PAQR6* expression (**Figure 4G**). These findings emphasize *PAQR6’s* role in shaping the immune microenvironment and its potential impact on immune-based therapies.

### 3.5 Construction of ceRNA network associated with *PAQR6*

**Figure 5:**
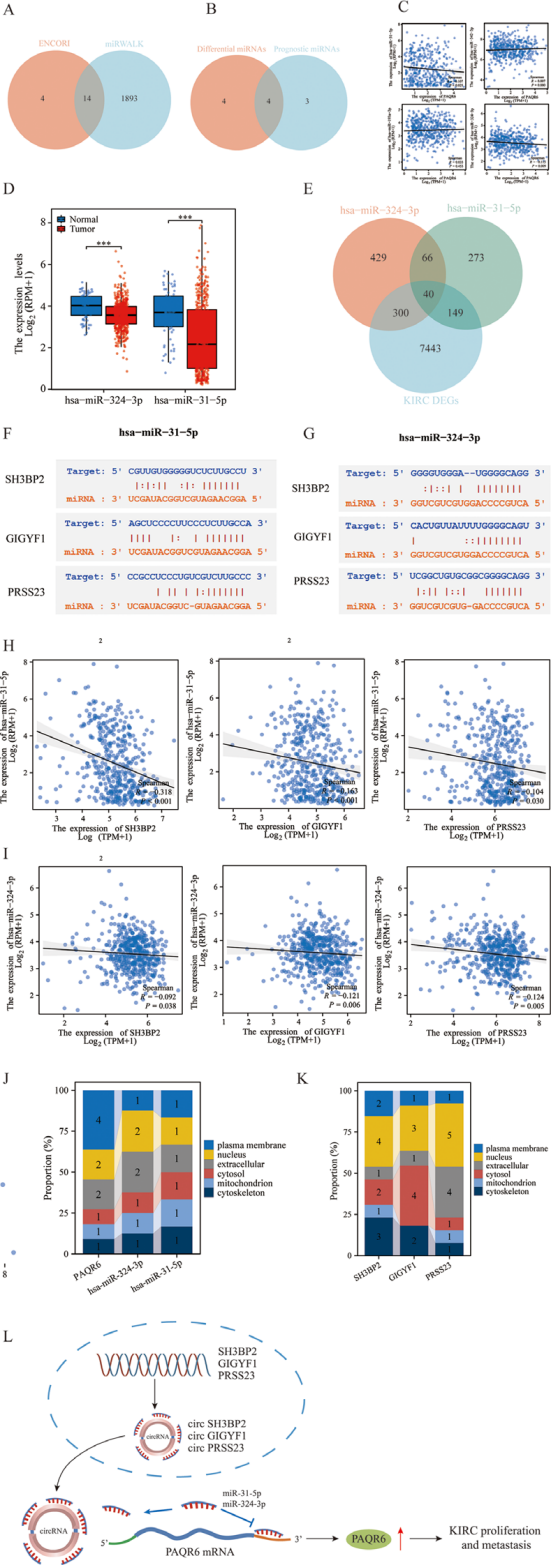
Construction of the ceRNA network related to the prognosis of KIRC. **(A)** Prediction of *PAQR6*-related miRNAs by the ENCORI and miRWALK databases. **(B)** Intersection of differential miRNAs related to *PAQR6* and prognostic miRNAs in KIRC. **(C)** Correlation of *PAQR6*-related miRNAs with differential prognosis of *PAQR6* in KIRC. **(D)** Expression of *PAQR6*-related miRNAs in KIRC. **(E)** Screening of targeted circRNAs for *PAQR6*-related miRNAs. **(F, G)** Gene sequences of miRNA-related circRNAs. **(H, I)** Correlation analysis of miRNA-related circRNAs and miRNAs. ***P < 0.001. **(J, K)** Localization of *PAQR6*, *PAQR6*-related miRNAs and circRANs in cells. **(L)** The possible carcinogenic mechanism of *PAQR6*.

Using the ENCORI database, we identify 18 miRNAs with potential targeting relationship to *PAQR6*, while the miRWALK database identified 1,907 such miRNAs. From these datasets, we identified 14 miRNAs that were common to both (**Figure 5A**). Among these 14 miRNAs, 4 were found to have prognostic differences in KIRC (**Figure 5B**). We analyzed the correlation between the miRNAs and *PAQR6* expression in KIRC, revealing a negative correlation between the miRNAs and their target gene. Specifically, hsa-miR-31-5p and hsa-miR-324-3p exhibited a strong negative correlation with *PAQR6* in KIRC (**Figure 5C**). Normal kidney tissues had higher levels of hsa-miR-31-5p and hsa-miR-324-3p expression than KIRC specimens (**Figure 5D**).

After examining circRNAs that target these miRNAs in more detail, we discovered 40 circRNAs that were expressed differently in KIRC samples than in normal kidney samples (**Figure 5E**). Among these, 3 circRNAs were associated with KIRC prognosis. Sequence information for hsa-miR-31-5p, hsa-miR-324-3p, and the three prognostic circRNAs was provided (**Figures 5F, G**). The correlation analysis confirmed a negative relationship between these circRNAs and hsa-miR-31-5p/hsa-miR-324-3p in KIRC (**Figures 5H, I**). We investigated the cellular localization of *PAQR6*, hsa-miR-324-3p, hsa-miR-31-5p, and the three circRNAs, finding that *PAQR6* is predominantly expressed on the plasma membrane (**Figures 5J, K**). Our analysis concluded that the prognosis of KIRC was connected with the ceRNA network including *PAQR6*-hsa-miR-31-5p/hsa-miR-324-3p-SH3BP2/GIGYF1/TRAK2/PRSS23. Finally, on the basis of the aforementioned research, we hypothesized the potential carcinogenic mechanism of *PAQR6*. The three circRNAs, SH3BP2, GIGYF1, and PRSS23, competitively bind to miR-31-5p and miR-324-3p, resulting in an elevation in the expression of *PAQR6*-related mRNA. Ultimately, this gives rise to the progression and invasion of KIRC (**Figure 5L**). Naturally, for a more precise carcinogenic mechanism, further investigations are requisite in the subsequent studies.

### 3.6 Therapeutic implications of *PAQR6* in KIRC

**Figure 6:**
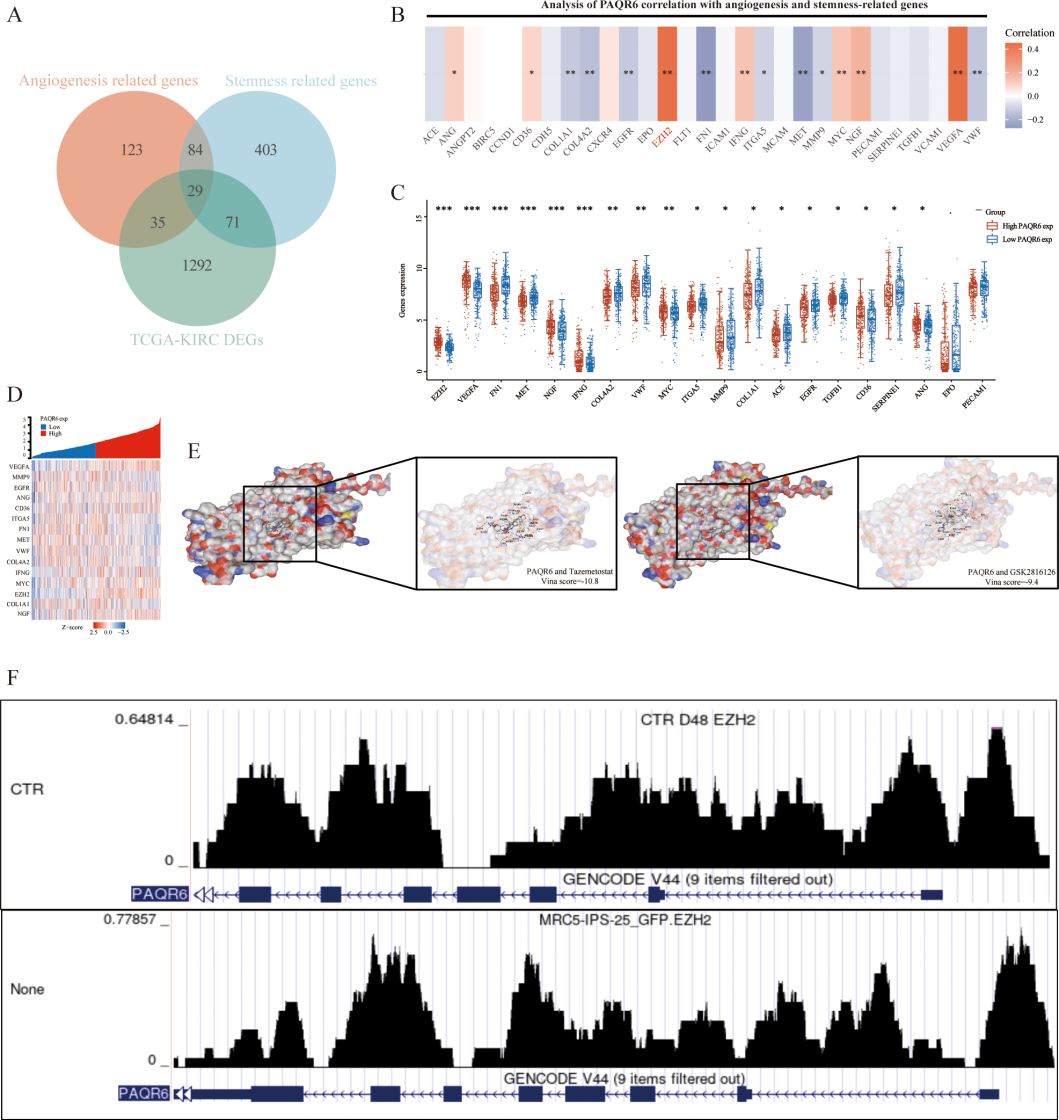
*PAQR6* is closely related to the oncogene EZH2. **(A)** Identification of angiogenesis and stem cell-related genes. **(B–D)** Analysis of angiogenesis and stemness-related genes related to *PAQR6*, and differential analysis of angiogenesis and stemness-related genes in the high-expression group and low-expression group of *PAQR6*. **(E)** Molecular docking of *PAQR6* with two EZH2 inhibitors. **(F)** EZH2 ChIP-seq Analysis of *PAQR6*. *: P<0.05; **: P<0.01; ***:P<0.001.

*PAQR6* may be involved in the angiogenesis and pluripotent stem cell differentiation pathways in KIRC cells, according to gene enrichment analysis. Using the Genecards database, we identified 29 key prognostic genes related to these pathways (**Figure 6A**). Among these, the expression of *PAQR6* and genes such as *ACE, ANGPT2, BIRC5, CCND1, CDH5, CXCR4, EPO, FLT1, ICAM1, MCAM, PECAM1, SERPINE1, TGFB1*, and *VCAM1* showed no significant correlation between high- and low-*PAQR6* expression groups. The strongest correlation was observed between *PAQR6* and EZH2 (**Figures 6B–D**). Considering *PAQR6*’s potential oncogenic effects through interaction with EZH2, we tested the binding affinity of two EZH2 inhibitors, Tazemetostat and GSK2816126, which are currently in clinical trials. Strong binding to *PAQR6* was demonstrated by both inhibitors (**Figure 6E**). EZH2, a critical transcription factor and well-known oncogene (27–29), plays a central role in regulating gene expression primarily through chromatin modifications. To further explore the transcriptional regulatory relationship between EZH2 and *PAQR6*, we performed ChIP-seq analysis. As shown in **Figure 6F**, significant EZH2 binding peaks were observed within the promoter and regulatory regions of *PAQR6* under control (CTR) conditions. In contrast, treatment (None) significantly altered these binding patterns, indicating a dynamic interaction between EZH2 and *PAQR6* under different conditions. These findings suggest that EZH2 may directly regulate *PAQR6* expression through epigenetic mechanisms, potentially linking this interaction to angiogenesis- and stemness-related pathways, thereby contributing to tumor progression. To further evaluate *PAQR6*’s potential as a therapeutic target for KIRC, we tested four angiogenesis-related drugs (sunitinib, sorafenib, pazopanib, and axitinib). The robust binding of these drugs to *PAQR6* (**Supplementary Figure S5**) underscores its role in angiogenesis and its promise as a potential target for KIRC therapy.

### 3.7 Experimental verification

**Figure 7:**
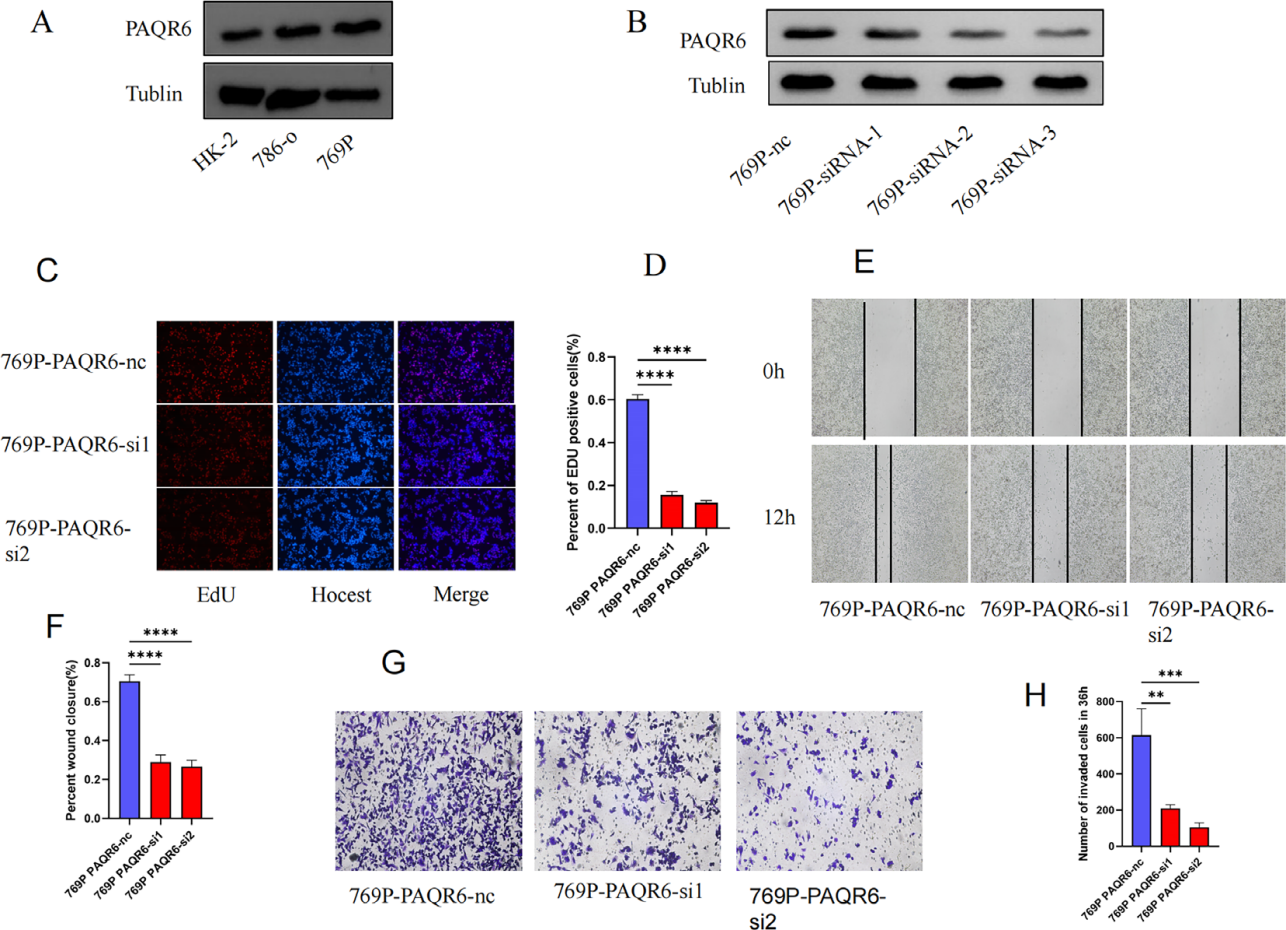
Functional verification of *PAQR6* in 769P cell. **(A)** The protein-level expression of *PAQR6* between normal and KIRC cell lines. **(B)** Verification of *PAQR6*-knockdown in 769P cells. **(C, D)** EDU experiment in *PAQR6*-knockdown 769P cells. The number of proliferating cells was calculated using the Image J software. **(E, F)** Scratch experiment in *PAQR6*-knockdown 769P cells. The area healed was quantified using the Image J software. **(G, H)** Transwell experiment in *PAQR6*-knockdown 769P cells. The number of invaded cells was calculated using the Image J software. All experiments were repeated at least three times, and the data were shown as means ± S.D. (**p < 0.01, ***P < 0.001, ****P < 0.0001).

Western blot was performed to evaluate the protein level of *PAQR6* in normal renal cell (HK-2) and KIRC cell lines (786-0 and 769P) (**Figure 7A**). The expression level of *PAQR6* was significantly increased in KIRC cell lines, particularly in 769P cell, compared with normal renal cell. Therefore,769P cell was selected to explore the role of *PAQR6* in KIRC phenotypes. The knockdown treatment was performed using three different siRNAs. Compared to the control, the protein level of *PAQR6* was significantly decreased in all three treatment groups (**Figure 7B**). The EdU assay was then performed to evaluate the impact of *PAQR6* on tumor cell proliferation. In the knockdown group, the decreased *PAQR6* significantly inhibited cell proliferation (**Figures 7C, D**). The scratch assay demonstrated that the knockdown of *PAQR6* evidently inhibited the migration of 769P cell (**Figures 7E, F**). Moreover, the number of invading cells was significantly reduced compared to the control group, demonstrating that the knockdown of *PAQR6* could inhibit cancer cell invasion (**Figures 7G, H**). These results indicated that the decreased *PAQR6* could inhibit the proliferation, migration and invasion of KIRC cells. These findings supported the hypothesis that targeting *PAQR6* could serve as a therapeutic strategy for inhibiting tumor growth and metastasis.

## 4 Discussion

*PAQR6*, a member of the PAQR family, is believed to respond to progesterone and is characterized by its ability to bind this hormone (30, 31). As a unique G protein-coupled receptor, *PAQR6* operates within the nervous system via the cAMP pathway (32). The human brain has significant levels of *PAQR6* expression, according to earlier research (13). *PAQR6* has been linked to the development of prostate and bladder cancer in the context of malignancies of the urinary system (21, 33, 42–45). However, its expression and function in KIRC have remained undefined. The present study aimed to explore the expression of *PAQR6* in KIRC, its prognostic significance, and its biological function using a variety of bioinformatics approaches, which were further validated through experimental methods.

Comparing our findings with prior studies, we demonstrate for the first time that *PAQR6* is significantly upregulated in KIRC and serves as a potential prognostic biomarker, with elevated expression correlating with poor overall survival and disease-specific survival. This is consistent with previous observations of *PAQR6* acting as a tumor-promoting factor in prostate and bladder cancer (21, 33). However, while earlier studies largely relied on gene expression data, we integrated multiple datasets (TCGA-KIRC and GSE15641) and performed experimental validation, strengthening the reliability of our conclusions.

Our study identifies a strong association between *PAQR6* and angiogenesis-related pathways, particularly through its correlation with EZH2, a well-known oncogene involved in cell cycle regulation and tumor progression (27, 28). EZH2 regulates pro-angiogenic factors such as *VEGFA* and *PDGFB* via epigenetic mechanisms (46, 47), and our findings suggest that *PAQR6* may modulate these processes through its interaction with EZH2. This transcriptional regulatory relationship is particularly relevant in KIRC, where angiogenesis plays a critical role in tumor progression and resistance to therapies like sunitinib (39, 40). Additionally, GSEA revealed significant enrichment of immune-related pathways associated with *PAQR6*, including Toll-like receptor signaling and hematopoietic stem cell pathways. The link between *PAQR6* and hypoxia-induced factors such as *HIF1A* suggests that it may act downstream of hypoxia-regulated pathways, integrating immune suppression and angiogenesis in the tumor microenvironment (34, 35).

*PAQR6’s* role in immune modulation was further supported by its correlation with specific immune cell types, such as monocytes, naive B cells, and CD8+ T cells. These immune cell types have been shown to influence cancer progression and prognosis in other contexts (36–38). Using the TIDE algorithm, we identified that high *PAQR6* expression is associated with poor responses to immune checkpoint inhibitors in high-risk patients, suggesting a novel therapeutic angle for improving immunotherapy outcomes. The dual role of *PAQR6* in modulating immune infiltration and angiogenesis makes it a promising target for therapeutic interventions in KIRC.

Compared to established KIRC biomarkers such as *VEGFA* and *CA9* (46, 48), *PAQR6* offers unique advantages. While *VEGFA* and *CA9* primarily focus on hypoxia-induced angiogenesis (39, 40), *PAQR6* integrates angiogenesis and immune modulation, providing a more comprehensive perspective. Additionally, its interaction with EZH2 introduces a novel regulatory mechanism not previously explored in KIRC, highlighting its distinct role in tumor progression and potential as a therapeutic target.

The ceRNA network constructed in this study highlights a novel regulatory mechanism involving *PAQR6*, two miRNAs (hsa-miR-31-5p and hsa-miR-324-3p), and four circular RNAs (circRNAs). These circRNAs may competitively bind to miRNAs, thereby increasing *PAQR6* expression and promoting tumor progression. This network not only sheds light on the post-transcriptional regulation of *PAQR6* but also aligns with previous research emphasizing the prognostic and functional significance of ceRNA networks in cancer (41). Although preliminary, these findings provide a foundation for further exploration of *PAQR6’s* regulatory networks.

To strengthen the clinical relevance of these findings, future research should include *in vivo* validation of *PAQR6’s* role in angiogenesis and immune modulation. Testing the efficacy of EZH2 inhibitors such as Tazemetostat in combination with existing anti-angiogenic therapies (e.g., sunitinib or axitinib) could provide insights into potential combination therapies for KIRC. Additionally, the development of *PAQR6*-specific inhibitors, guided by molecular docking and structural modeling, could further enhance its therapeutic potential.

In summary, our study provides novel insights into the role of *PAQR6* in KIRC and its interaction with key pathways, immune cells, and angiogenesis-related processes. Unlike prior research, we combined bioinformatics analyses, molecular docking, and experimental validation to offer a more comprehensive perspective. However, further studies are necessary to validate these findings in larger cohorts and to explore the therapeutic potential of targeting *PAQR6* in KIRC.

## 5 Conclusion

This study confirmed the distinct expression of *PAQR6* in KIRC, highlighting its potential as a prognostic biomarker for this disease. Our findings also revealed the intricate interaction between *PAQR6* and EZH2, suggesting their role in regulating angiogenesis and pluripotent stem cell differentiation pathways in KIRC cells. Furthermore, the identification of specific ceRNA networks provides a foundation for potential therapeutic interventions in KIRC. However, further experiments are necessary to investigate the molecular mechanisms underlying the function of *PAQR6* in KIRC.

## Data Availability

Publicly available datasets were analyzed in this study. This data can be found here: TCGA-TCGA: https://portal.gdc.cancer.gov/GEO (GSE15641,GSE29609): https://www.ncbi.nlm.nih.gov/geo/.

## Supplementary material

Supplementary Figure 1 The screening process of the *PAQR6* gene.

Supplementary Figure 2 Immune cell types significantly related to KIRC.

Supplementary Figure 3 Establishment of the immune cell prediction model.

Supplementary Figure 4 Correlation of the prognostic model with immune stimulants and immunosuppressants.

Supplementary Figure 5 Strong binding of four drugs to *PAQR6*.

Supplementary Table 1 The clinical characteristics of TCGA-KIRC patients included in this study.

Supplementary Table 2 The clinical characteristics of GSE15641 patients included in this study.

Supplementary Table 3 The clinical characteristics of GSE29609 patients included in this study. Antibodies: Information on antibodies used in Western Blot experiments. Flowchart: The bioinformatics analysis flow chart used in this article.

Supplementary Table 4 The clinical characteristics of GSE111360 patients included in this study.

## References

[1] Shen D Ding L Lu Z Wang R Yu C Wang H. Mettl14-Mediated Lnc-Lsg1 M6a modification inhibits clear cell renal cell carcinoma metastasis via regulating Esrp2 ubiquitination. Mol Ther Nucleic Acids. (2022) 27:547–61. doi: 10.1016/j.omtn.2021.12.024 PMC8738955 35036065

[2] Bai D Feng H Yang J Yin A Lin X Qian A. Genomic analysis uncovers prognostic and immunogenic characteristics of ferroptosis for clear cell renal cell carcinoma. Mol Ther Nucleic Acids. (2021) 25:186–97. doi: 10.1016/j.omtn.2021.05.009 PMC8368772 34458004

[3] Xu Y Lv D Yan C Su H Zhang X Shi Y. Mettl3 promotes lung adenocarcinoma tumor growth and inhibits ferroptosis by stabilizing Slc7a11 M(6)a modification. Cancer Cell Int. (2022) 22:11. doi: 10.1186/s12935-021-02433-6 34996469 PMC8742440

[4] Flanigan RC Mickisch G Sylvester R Tangen C Van Poppel H Crawford ED. Cytoreductive nephrectomy in patients with metastatic renal cancer: a combinedanalysis. J Urol. (2004) 171:1071–6. doi: 10.1097/01.ju.0000110610.61545.ae 14767273

[5] Bex A Albiges L Ljungberg B Bensalah K Dabestani S Giles RH. Updated European Association of Urology guidelines for cytoreductive nephrectomy in patients with synchronous metastatic clear-cell renal cell carcinoma. Eur Urol. (2018) 74:805–9. doi: 10.1016/j.eururo.2018.08.008 30177291

[6] Flippot R Escudier B Albiges L. Immune checkpoint inhibitors: toward new paradigms in renal cell carcinoma. Drugs. (2018) 78:1443–57. doi: 10.1007/s40265-018-0970-y 30187355

[7] Motzer RJ Tannir NM McDermott DF Arén Frontera O Melichar B Choueiri TK. Nivolumab plus ipilimumab versus sunitinib in advanced renal-cell carcinoma. N Engl J Med. (2018) 378:1277–90. doi: 10.1056/NEJMoa1712126 PMC5972549 29562145

[8] Hsieh JJ Purdue MP Signoretti S Swanton C Albiges L Schmidinger M. Renal cell carcinoma. Nat Rev Dis Primers. (2017) 3:1–9. doi: 10.1038/nrdp.2017.9 PMC5936048 28276433

[9] Monteiro FS Soares A Rizzo A Santoni M Mollica V Grande E. The role of immune checkpoint inhibitors (ICI) as adjuvant treatment in renal cell carcinoma (RCC): A systematic review and meta-analysis. Clin Genitourin Cancer. (2023) 21:324–33. doi: 10.1016/j.clgc.2023.01.005 36823017

[10] Norberg SM Hinrichs CS. Engineered T cell therapy for viral and non-viral epithelial cancers. Cancer Cell. (2023) 41:58–69. doi: 10.1016/j.ccell.2022.10.016 36400016 PMC9839504

[11] Soerens AG Künzli M Quarnstrom CF Scott MC Swanson L Locquiao JJ. Functional T cells are capable of supernumerary cell division and longevity. Nature. (2023) 614:762–6. doi: 10.1038/s41586-022-05626-9 PMC11617068 36653453

[12] Bell HN Huber AK Singhal R Korimerla N Rebernick RJ Kumar R. Microenvironmental ammonia enhances T cell exhaustion in colorectal cancer. Cell Metab. (2023) 35:134–49. doi: 10.1016/j.cmet.2022.11.013 PMC9841369 36528023

[13] Tang YT Hu T Arterburn M Boyle B Bright JM Emtage PC. PAQR proteins: a novel membrane receptor family defined by an ancient7-transmembrane pass motif. J Mol Evol. (2005) 61:372–80. doi: 10.1007/s00239-004-0375-2 16044242

[14] Valadez-Cosmes P Vázquez-Martínez ER Cerbon M Camacho-Arroyo I. Membrane progesterone receptors in reproduction and cancer. Mol Cell Endocrinol. (2016) 434:166–75. doi: 10.1016/j.mce.2016.06.027 27368976

[15] Tian L Luo N Zhu X Chung BH Garvey WT Fu Y. Adiponectin-AdipoR1/2-APPL1 signaling axis suppresses human foam cell formation: differential ability of AdipoR1 and AdipoR2 to regulate inflammatory cytokine responses. Atherosclerosis. (2012) 221:66–75. doi: 10.1016/j.atherosclerosis.2011.12.014 22227293 PMC3288755

[16] Lei L Ling ZN Chen XL Hong LL Ling ZQ. Characterization of the Golgi scaffold protein PAQR3, and its role in tumor suppression and metabolic pathway compartmentalization. Cancer Manag Res. (2020) 12:353–62. doi: 10.2147/CMAR.S210919 PMC6970510 32021448

[17] Sinreih M Knific T Thomas P Grazio SF Rižner TL. Membrane progesterone receptors β and γ have potential as prognostic biomarkers of endometrial cancer. J Steroid Biochem Mol Biol. (2018) 178:303–11. doi: 10.1016/j.jsbmb.2018.01.011 29353001

[18] Romero-Sánchez M Peiper SC Evans B Wang Z Catasús L Ribe A. Expression profile of heptahelical putative membrane progesterone receptors in epithelial ovarian tumors. Hum Pathol. (2008) 39:1026–33. doi: 10.1016/j.humpath.2007.11.007 18479732

[19] González-Orozco JC Hansberg-Pastor V Valadez-Cosmes P Nicolas-Ortega W Bastida-Beristain Y Fuente-Granada M. Activation of membrane progesterone receptor-alpha increases proliferation, migration, and invasion of human glioblastoma cells. Mol Cell Endocrinol. (2018) 477:81–9. doi: 10.1016/j.mce.2018.06.004 29894708

[20] Zhou L Zhou W Zhang H Hu Y Yu L Zhang Y. Progesterone suppresses triple-negative breast cancer growth and metastasis to the brain via membrane progesterone receptor α. Int J Mol Med. (2017) 40:755–61. doi: 10.3892/ijmm.2017.3060 PMC5548012 28713912

[21] Li B Lin Z Liang Q Hu Y Xu WF. PAQR6 expression enhancement suggests a worse prognosis in prostate cancer patients. Open Life Sci. (2018) 13:511–7. doi: 10.1515/biol-2018-0061 PMC7874734 33817121

[22] Wang Y Ma L He J Gu H Zhu H. Identification of cancer stem cell-related genes through single cells and machine learning for predicting prostate cancer prognosis and immunotherapy. Front Immunol. (2024) 15:1464698. doi: 10.3389/fimmu.2024.1464698 39267762 PMC11390519

[23] Wang Y He J Zhao Q Bo J Zhou Y Sun H. Evaluating the predictive value of angiogenesis-related genes for prognosis and immunotherapy response in prostate adenocarcinoma using machine learning and experimental approaches. Front Immunol. (2024) 15:1416914. doi: 10.3389/fimmu.2024.1416914 38817605 PMC11137278

[24] Hao H Wang Z Ren S Shen H Xian H Ge W. Reduced GRAMD1C expression correlates to poor prognosis and immune infiltrates in kidney renal clear cell carcinoma. PeerJ. (2019) 7:e8205. doi: 10.7717/peerj.8205 31875150 PMC6927341

[25] Li JH Liu S Zhou H Qu LH Yang JH. starBase v2.0: decoding miRNA-ceRNA, miRNA-ncRNA and protein-RNA interaction networks from large-scale CLIP-Seq data. Nucleic Acids Res. (2014) 42:D92–7. doi: 10.1093/nar/gkt1248 PMC3964941 24297251

[26] Karagkouni D Paraskevopoulou MD Chatzopoulos S Vlachos IS Tastsoglou S Kanellos I. DIANA-TarBase v8: a decade-long collection of experimentally supported miRNA-gene interactions. Nucleic Acids Res. (2018) 46:D239–45. doi: 10.1093/nar/gkx1141 PMC5753203 29156006

[27] Duan R Du W Guo W. EZH2: a novel target for cancer treatment. J Hematol Oncol. (2020) 13:104. doi: 10.1186/s13045-020-00937-8 32723346 PMC7385862

[28] Zhang DY Sun QC Zou XJ Song Y Li WW Guo ZQ. Long noncoding RNA UPK1A-AS1 indicates poor prognosis of hepatocellular carcinoma and promotes cell proliferation through interaction with EZH2. J Exp Clin Cancer Res. (2020) 39:229. doi: 10.1186/s13046-020-01748-y 33121524 PMC7596966

[29] Xiao G Jin LL Liu CQ Wang YC Meng YM Zhou ZG. EZH2 negatively regulates PD-L1 expression in hepatocellular carcinoma. J Immunother Cancer. (2019) 7:300. doi: 10.1186/s40425-019-0784-9 31727135 PMC6854886

[30] Smith JL Kupchak BR Garitaonandia I Hoang LK Maina AS Regalla LM. Heterologous expression of human mPRalpha, mPRbeta and mPRgamma in yeast confirms their ability to function as membrane progesterone receptors. Steroids. (2008) 73:1160–73. doi: 10.1016/j.steroids.2008.05.003 PMC2597464 18603275

[31] Zhao X Mo D Li A Gong W Zhang Y Qian W. Characterization and transcriptional regulation analysis of the porcine PAQR6 gene. DNA Cell Biol. (2011) 30:947–54. doi: 10.1089/dna.2011.1262 21988462

[32] Thomas P Pang Y. Membrane progesterone receptors: evidence for neuroprotective, neurosteroid signaling and neuroendocrine functions in neuronal cells. Neuroendocrinology. (2012) 96:162–71. doi: 10.1159/000339822 PMC3489003 22687885

[33] Cai Z Chen H Bai J Zheng Y Ma J Cai X. Copy number variations of CEP63, FOSL2 and PAQR6 serve as novel signatures for the prognosis of bladder cancer. Front Oncol. (2021) 11:674933. doi: 10.3389/fonc.2021.674933 34041036 PMC8141655

[34] Ren S Wang W Shen H Zhang C Hao H Sun M. Development and validation of a clinical prognostic model based on immune-related genes expressed in clear cell renal cell carcinoma. Front Oncol. (2020) 10:1496. doi: 10.3389/fonc.2020.01496 32983989 PMC7485294

[35] Guo SB Pan DQ Su N Huang MQ Zhou ZZ Huang WJ. Comprehensive scientometrics and visualization study profiles lymphoma metabolism and identifies its significant research signatures. Front Endocrinol (Lausanne). (2023) 14:1266721. doi: 10.3389/fendo.2023.1266721 37822596 PMC10562636

[36] Li XL Xue Y Yang YJ Zhang CX Wang Y Duan YY. Hematopoietic stem cells: cancer involvement and myeloid leukemia. Eur Rev Med Pharmacol Sci. (2015) 19:1829–36. 26044227

[37] Lee YY Choi CH Sung CO Do IG Huh S Song T. Prognostic value of pre-treatment circulating monocyte count in patients with cervical cancer: comparison with SCC-Ag level. Gynecol Oncol. (2012) 124:92–7. doi: 10.1016/j.ygyno.2011.09.034 22014631

[38] Wilcox RA Ristow K Habermann TM Inwards DJ Micallef IN Johnston PB. The absolute monocyte and lymphocyte prognostic score predicts survival and identifies high-risk patients in diffuse large-B-cell SEMoma. Leukemia. (2011) 25:1502–9. doi: 10.1038/leu.2011.112 21606957

[39] Hou P Li H Yong H Chen F Chu S Zheng J. PinX1 represses renal cancer angiogenesis via the mir-125a-3p/VEGF signaling pathway. Angiogenesis. (2019) 22:507–19. doi: 10.1007/s10456-019-09675-z 31254127

[40] Chen Y Lu Z Qi C Yu C Li Y Huan W. N6-methyladenosine-modified TRAF1 promotes sunitinib resistance by regulating apoptosis and angiogenesis in a METTL14-dependent manner in renal cell carcinoma. Mol Cancer. (2022) 21:111. doi: 10.1186/s12943-022-01549-1 35538475 PMC9087993

[41] Lu J Kang X Wang Z Zhao G Jiang B. The activity level of follicular helper T cells in the peripheral blood of osteosarcoma patients is associated with poor prognosis. Bioengineered. (2022) 13:3751–9. doi: 10.1080/21655979.2022.2031387 PMC8974108 35081874

[42] Wang Y Wang J Zhang L He J Ji B Wang J. Unveiling the role of YARS1 in bladder cancer: A prognostic biomarker and therapeutic target. J Cell Mol Med. (2024) 28:1–20. doi: 10.1111/jcmm.18213 PMC10951887 38506098

[43] Wang Y Wang J Liu Y Wang X Ren M. Multidimensional pan-cancer analysis of HSPA5 and its validation in the prognostic value of bladder cancer. Heliyon. (2024) 10:e27184. doi: 10.1016/j.heliyon.2024.e27184 38496902 PMC10944199

[44] Pang ZQ Wang JS Wang JF Wang YX Ji B Xu YD. JAM3: A prognostic biomarker for bladder cancer via epithelial-mesenchymal transition regulation. Biomol Biomed. (2024) 24:897–911. doi: 10.17305/bb.2024.9979 38400838 PMC11293228

[45] Wang Y Ji B Zhang L Wang J He J Ding B. Identification of metastasis-related genes for predicting prostate cancer diagnosis, metastasis and immunotherapy drug candidates using machine learning approaches. Biol Direct. (2024) 19:50. doi: 10.1186/s13062-024-00494-x 38918844 PMC11197330

[46] Hicklin DJ Ellis LM. Role of the vascular endothelial growth factor pathway in tumor growth and angiogenesis. J Clin Oncol. (2005) 23:1011–27. doi: 10.1200/JCO.2005.06.081 15585754

[47] Demoulin JB Essaghir A. PDGF receptor signaling networks in normal and cancer cells. Cytokine Growth Factor Rev. (2014) 25:273–83. doi: 10.1016/j.cytogfr.2014.03.003 24703957

[48] Pastorekova S Zatovicova M Pastorek J. Cancer-associated carbonic anhydrases and their inhibition. Curr Pharm Des. (2008) 14:685–98. doi: 10.2174/138161208783877893 18336315

