# Supplementary material for: PAQR6 as a prognostic biomarker and potential therapeutic target in kidney renal clear cell carcinoma
Source: Front Immunol. 2024 Dec 17;15:1521629. doi: 10.3389/fimmu.2024.1521629 (PMC11685228; doi:10.3389/fimmu.2024.1521629)
Supplement: Supplementary Figure 1 — The screening process of the PAQR6 gene. [file DataSheet1.zip › Supplementary material/Antibodies.pdf]

Anti-PAQR6 Antibody

Catalog Number: ***A14355-1***

antibody and ELISA experts
BOSTER BIOLOGICAL TECHNOLOGY
Building C21, 3rd and 4th floors, Optics Valley Biomedical Accelerator,
Wuhan East Lake High-tech Development Zone
Web: www.boster.com ***Phone:*** 027-67845390 ***Email:*** boster@boster.com

Basic Information

**Product Name**	Anti-PAQR6 Antibody
**Gene Name**	PAQR6
**Source**	Rabbit
**Clonality**	Polyclonal
**Isotype**	IgG
**Species Reactivity**	human, mouse, rat
**Tested Application**	WB, ELISA
**Contents**	500 ug/ml antibody with PBS, 0.02% NaN3, 1 mg/ml BSA and 50% glycerol.
**Immunogen**	E.coli-derived human PAQR6 recombinant protein (Position: M1-A292).
**Concentration**	500 ug/ml
**Purification**	Immunogen affinity purified.
**Observed MW**	38 kDa
**Dilution Ratios**	Western blot (WB): 1:500-2000<br>Enzyme linked immunosorbent assay (ELISA):1:100-1000

Storage

12 months from date of receipt，-20℃ as supplied. 6 months 2 to 8℃ after reconstitution. Avoid repeated freezing and thawing.

Background Information

Membrane progesterone receptor delta (mPRδ), or progestin and adipoQ receptor 6 (PAQR6), is a protein that in humans is encoded by the PAQR6 gene. Predicted to enable signaling receptor activity. Predicted to be located in plasma membrane. Predicted to be integral component of membrane.

Selected Validation Data

antibody and ELISA experts
BOSTER BIOLOGICAL TECHNOLOGY
Building C21, 3rd and 4th floors, Optics Valley Biomedical Accelerator,
Wuhan East Lake High-tech Development Zone
Web: www.boster.com **Phone:** 027-67845390 **Email:** boster@boster.com

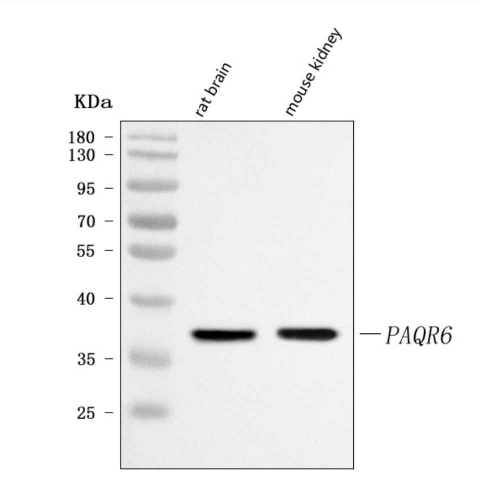

Figure 1. Western blot analysis of anti-PAQR6 antibody (A14355-1). The sample well of each lane was loaded with 30 ug of sample under reducing conditions.
Lane 1: rat brain tissue lysates,
Lane 2: mouse kidney tissue lysates.
After electrophoresis, proteins were transferred to a membrane. Then the membrane was incubated with rabbit anti-PAQR6 antigen affinity purified polyclonal antibody (A14355-1) and probed with a goat anti-rabbit IgG-HRP secondary antibody (Catalog # BA1054). The signal is developed using ECL Plus Western Blotting Substrate (Catalog # AR1197). A specific band was detected for PAQR6 at approximately 38 kDa. The expected band size for PAQR6 is at 38 kDa.

FOR RESEARCH USE ONLY. NOT FOR DIAGNOSTIC AND CLINICAL USE
