# Supplementary material for: PAQR6 as a prognostic biomarker and potential therapeutic target in kidney renal clear cell carcinoma
Source: Front Immunol. 2024 Dec 17;15:1521629. doi: 10.3389/fimmu.2024.1521629 (PMC11685228; doi:10.3389/fimmu.2024.1521629)
Supplement: Supplementary Figure 1 — The screening process of the PAQR6 gene. [file DataSheet1.zip › Supplementary material/Supplementary Figure.docx]

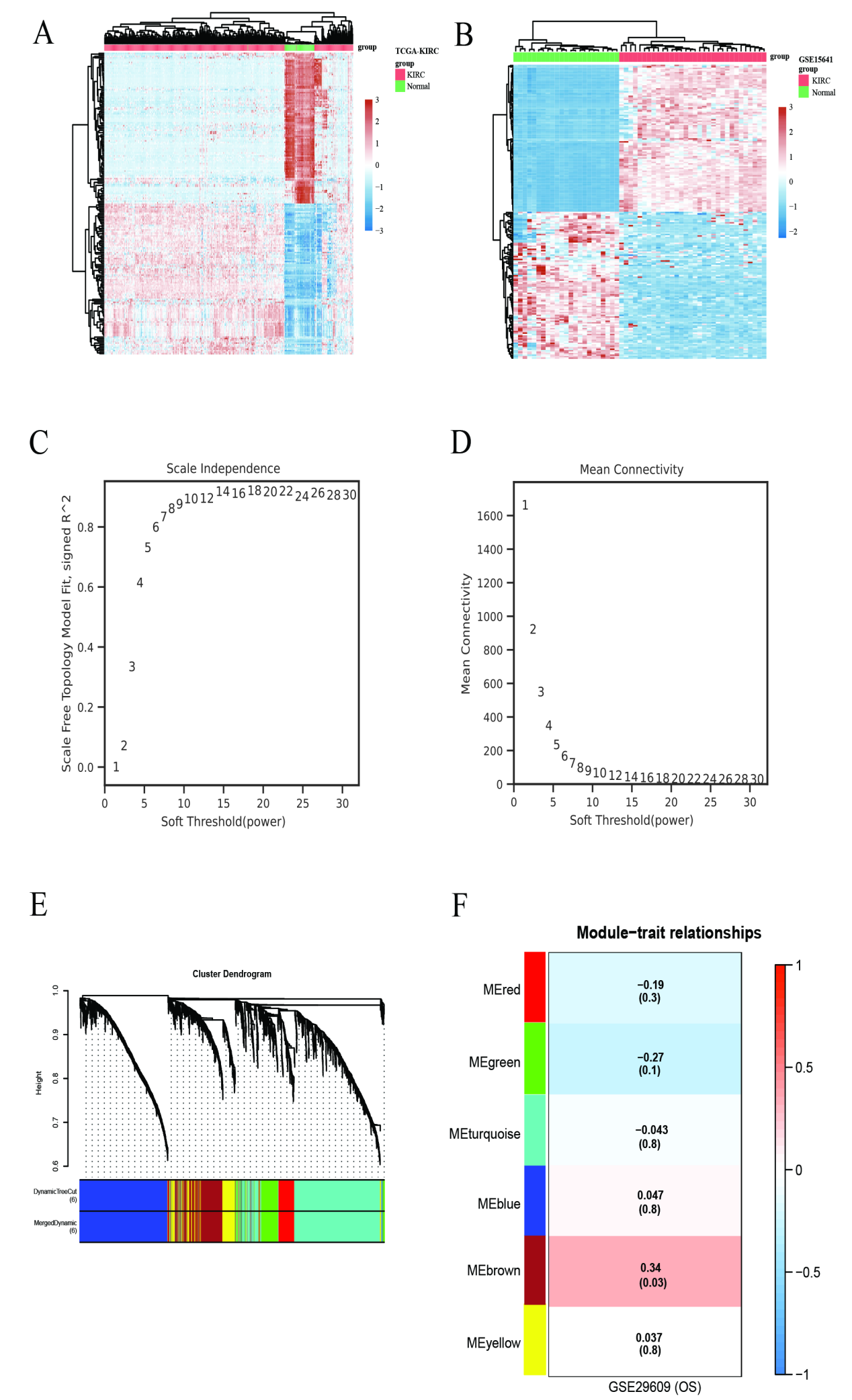


FIGURE S1: **Differential expression analysis and WGCNA**. (A) Heatmap of differentially expressed genes (DEGs) in TCGA-KIRC dataset. (B) Heatmap of differentially expressed genes (DEGs) in GSE15641 dataset. (C) Analysis of scale independence for soft-thresholding power in WGCNA. (D) Analysis of mean connectivity for soft-thresholding power in WGCNA. (E) Cluster dendrogram and module assignment in WGCNA. (F) Module-trait relationships.


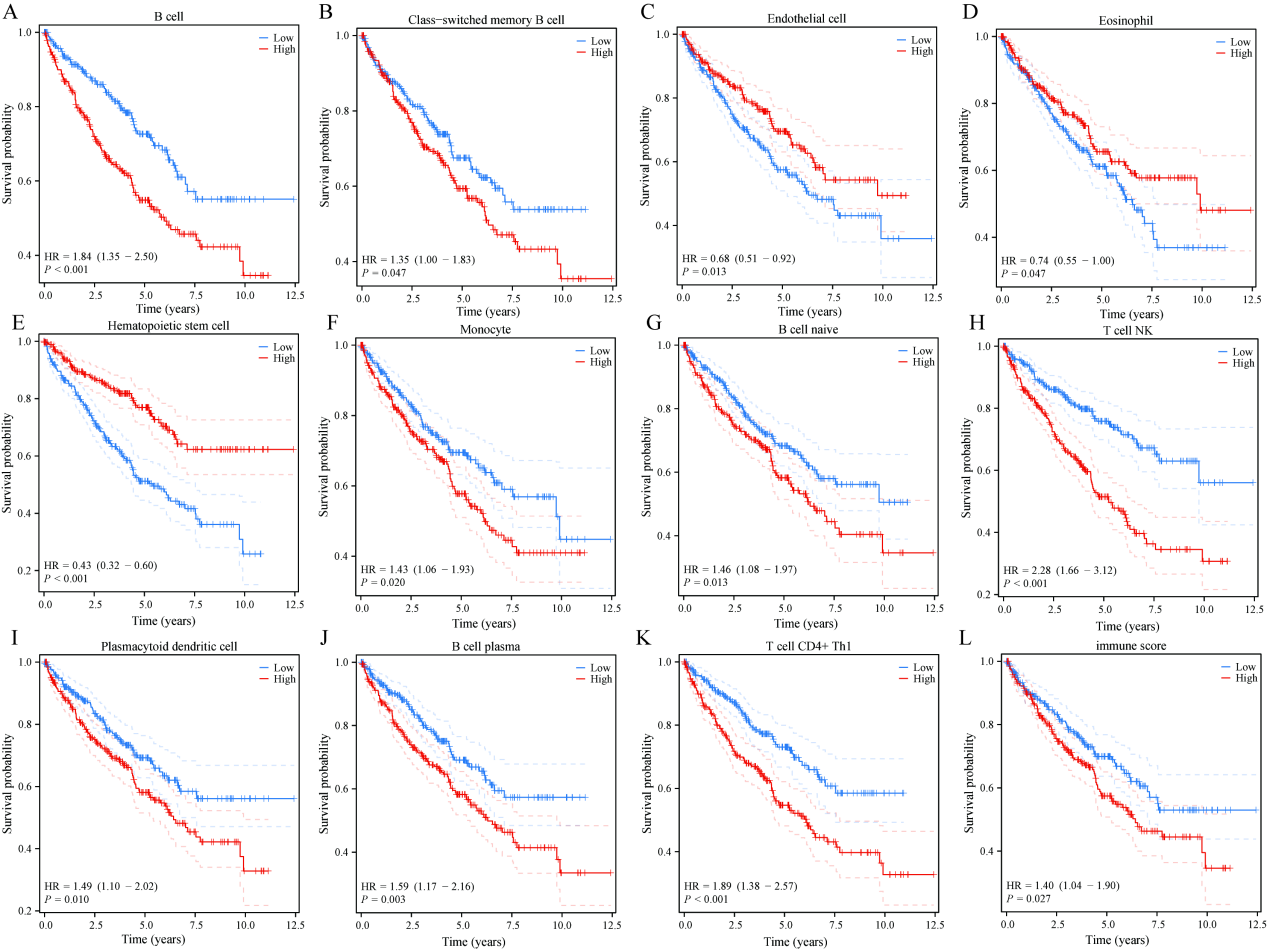


Figure S2: Immune cell types significantly related to KIRC.
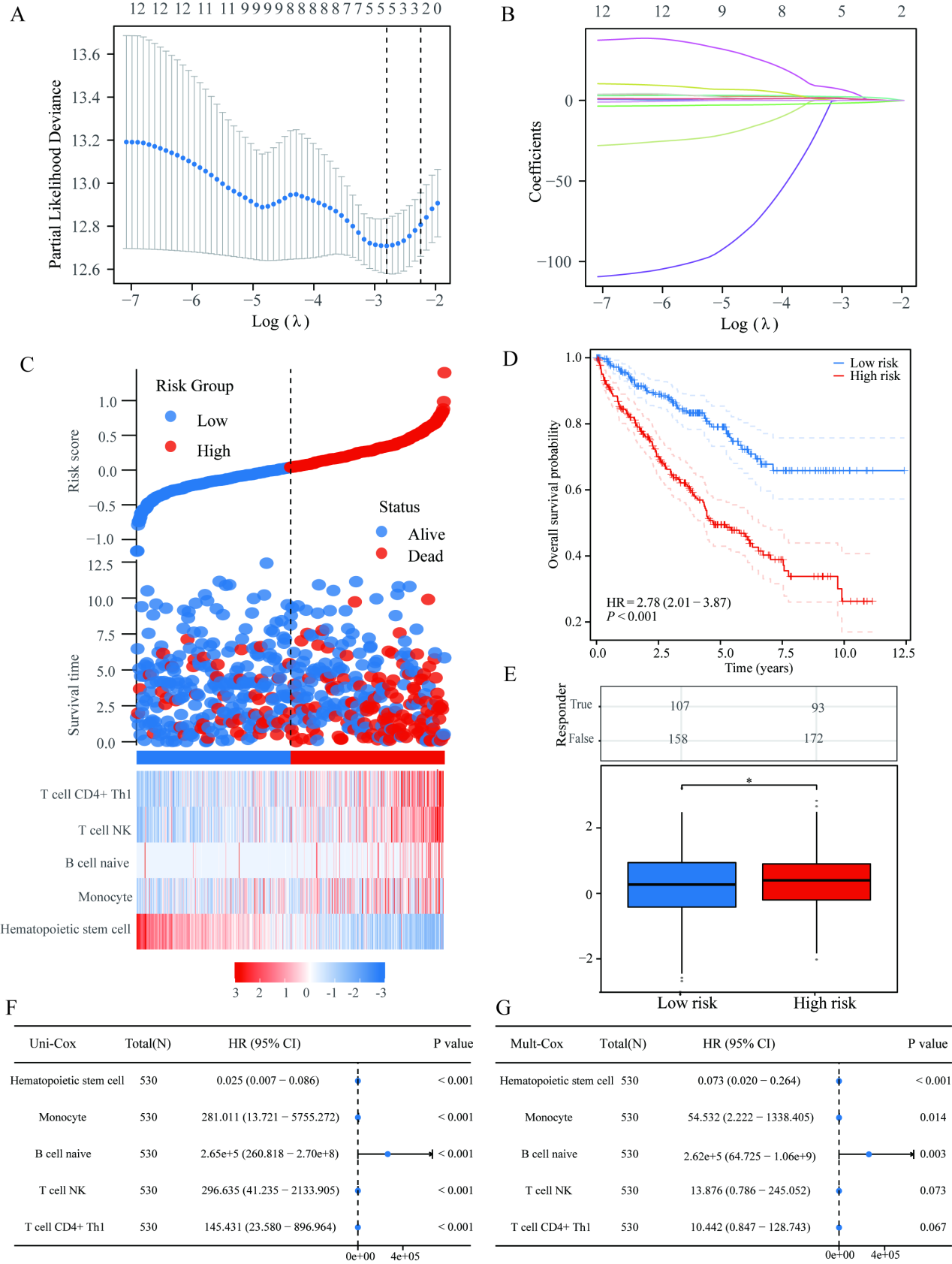


Figure S3: Establishment of the immune cell prediction model.(A) Twelve immune cells were identified using the LASSO technique. (B) Five immune cells were included in the prediction model. (C) Five predictive cells related to immune infiltration and their expression patterns were confirmed in KIRC patients with different risk scores and different survival outcomes. (D) The KM curve shows that the participants were divided into high-risk and low-risk groups. (E) Evaluation of the reactivity of immune checkpoint inhibitors in high-risk and low-risk populations. (F) The prognostic significance of five different immune cell types was tested by univariate Cox regression analysis. (G) The prognostic significance of five different immune cell types was evaluated by multivariate Cox regression analysis. *P < 0.05.


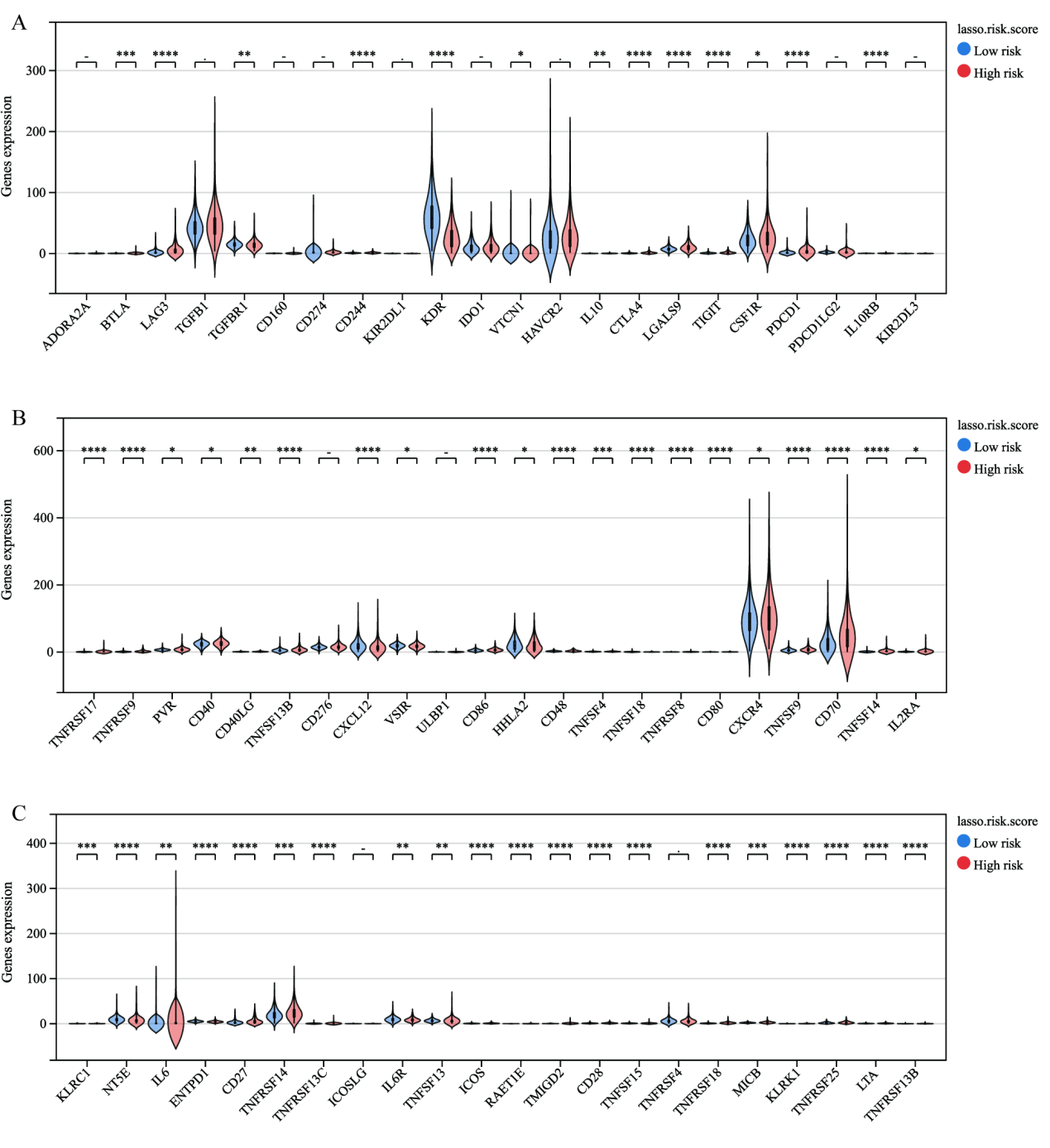


Figure S4: Correlation of the prognostic model with immune stimulants and immunosuppressants. （A-C）Correlation of the prognostic model with immune stimulants and immunosuppressants. *P < 0.05, **p < 0.01, ***P < 0.001, ****P < 0.0001.


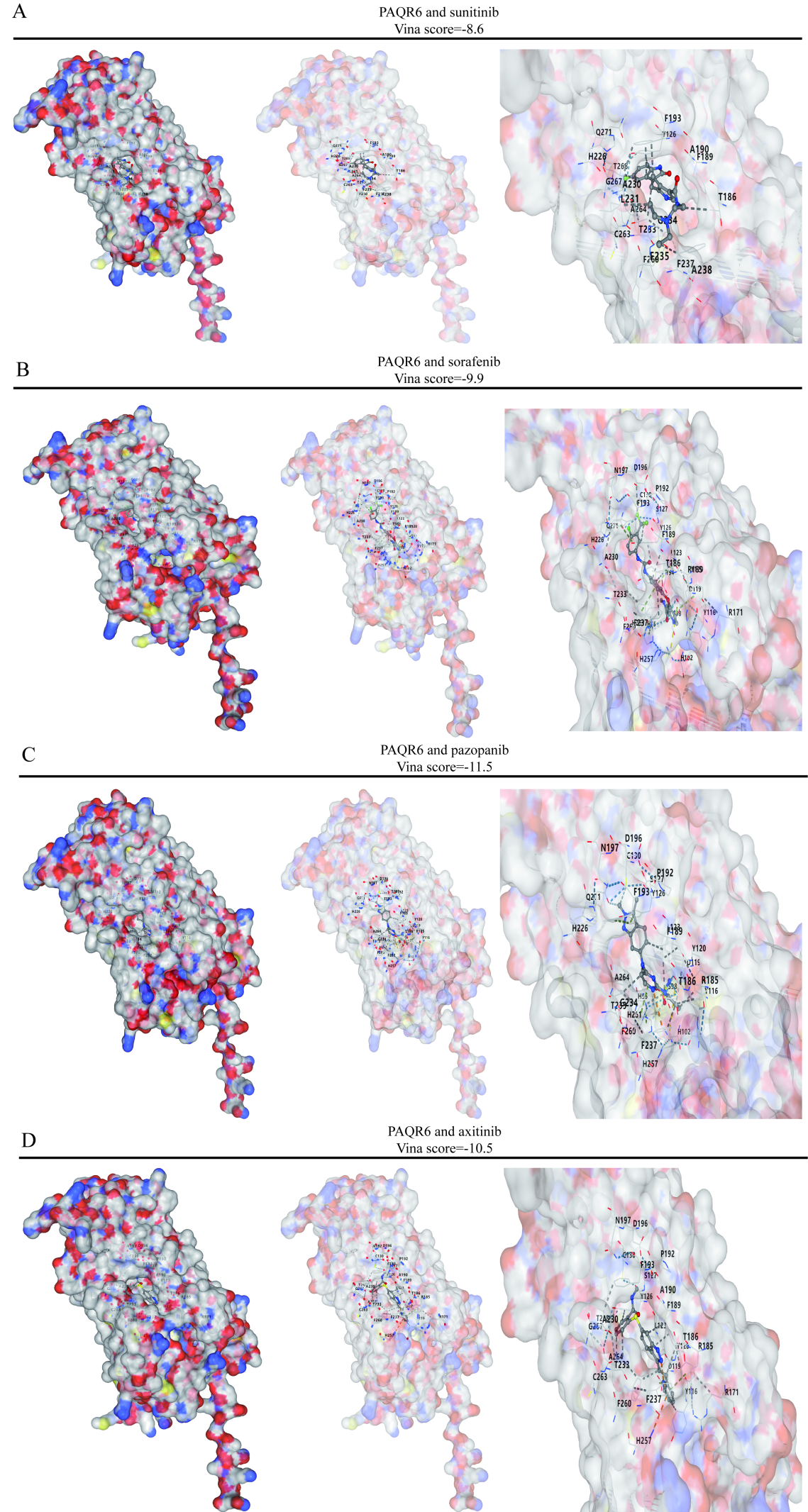


Figure S5: Strong binding of four drugs to *PAQR6*.
