# Supplementary figures and images for: PAQR6 as a prognostic biomarker and potential therapeutic target in kidney renal clear cell carcinoma
Source: Front Immunol. 2024 Dec 17;15:1521629. doi: 10.3389/fimmu.2024.1521629 (PMC11685228; doi:10.3389/fimmu.2024.1521629)

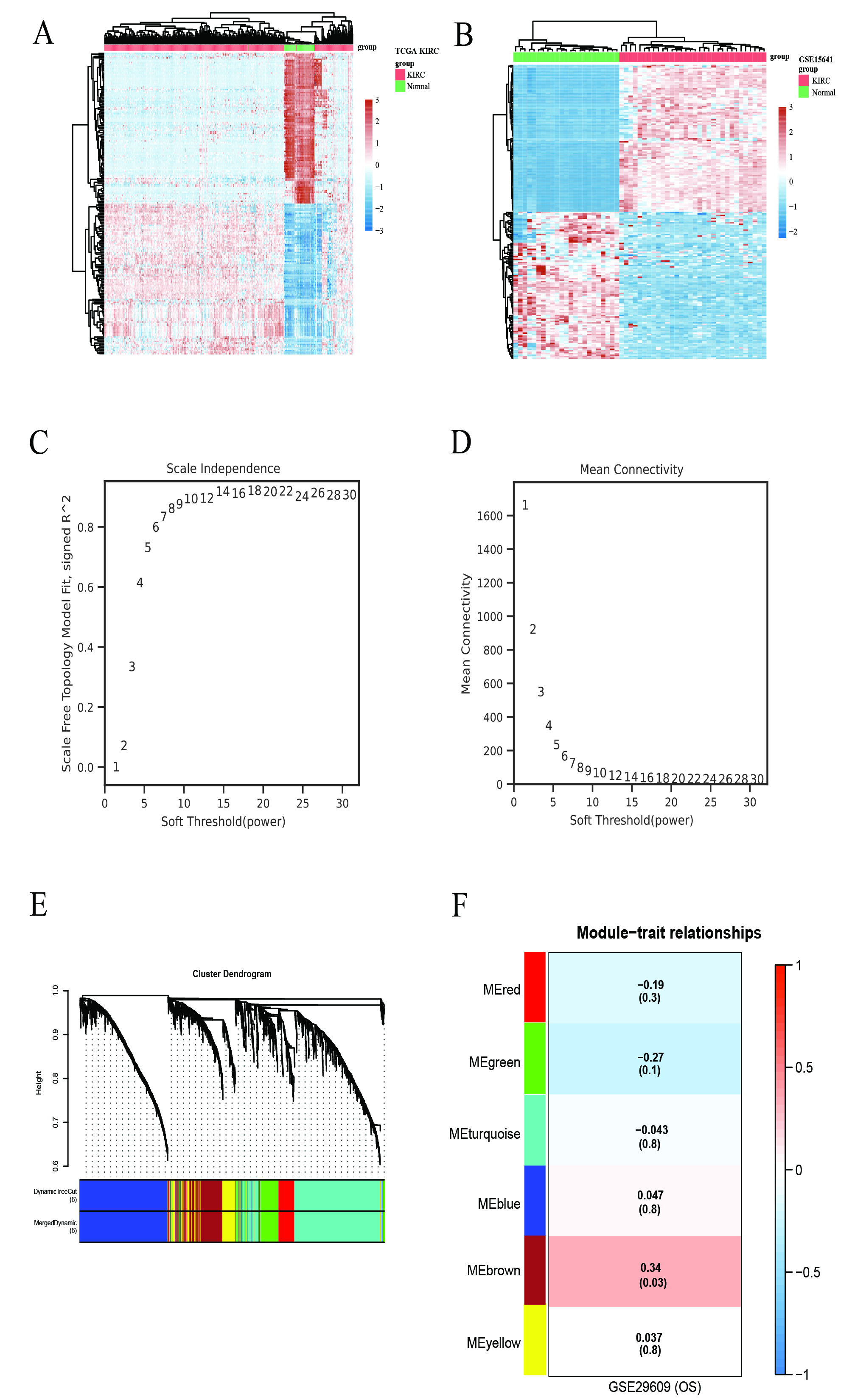

Supplement: Supplementary Figure 1 — The screening process of the PAQR6 gene. [file DataSheet1.zip › Supplementary material/Fig S1.tif]

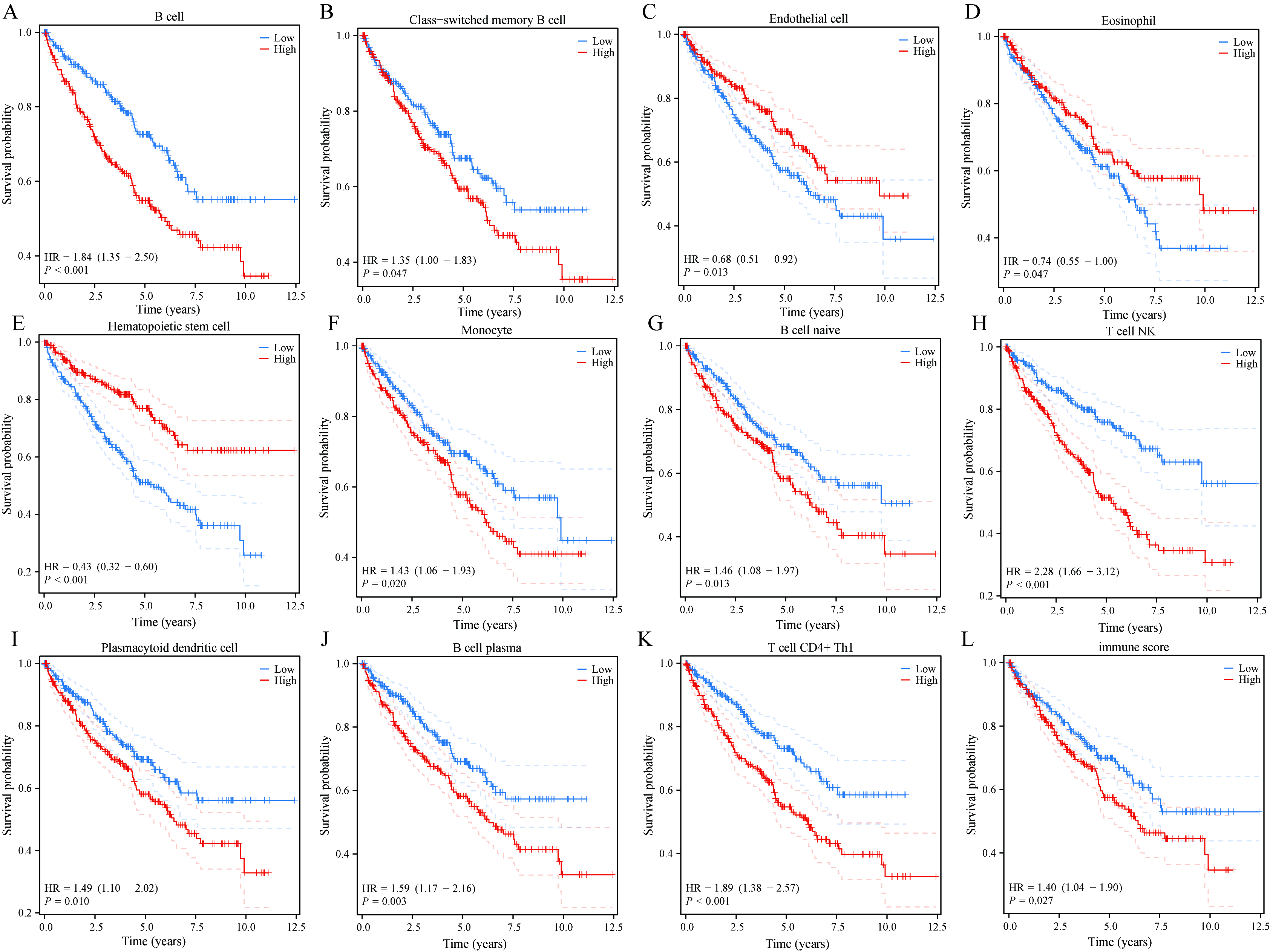

Supplement: Supplementary Figure 1 — The screening process of the PAQR6 gene. [file DataSheet1.zip › Supplementary material/Fig S2.tif]

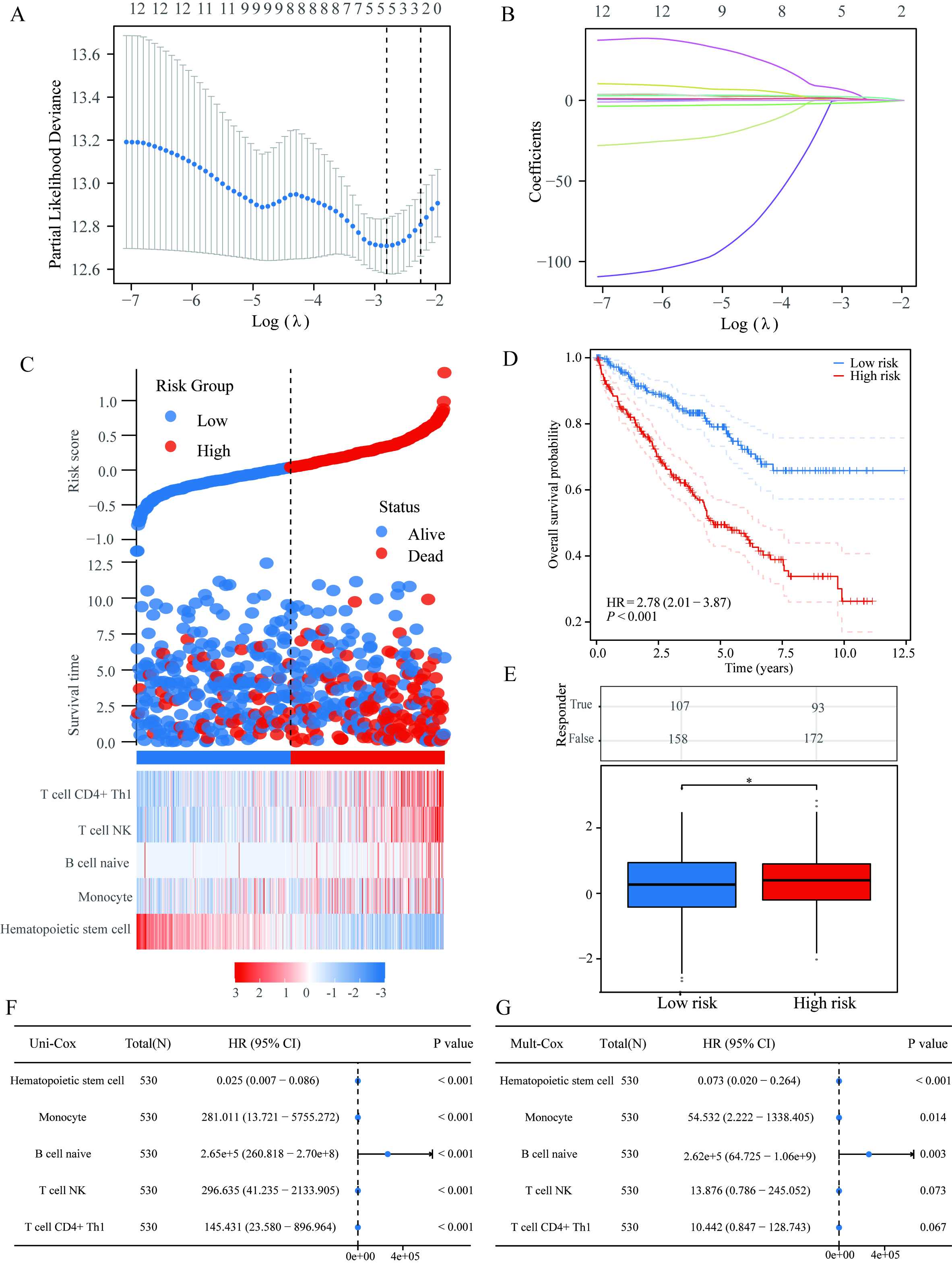

Supplement: Supplementary Figure 1 — The screening process of the PAQR6 gene. [file DataSheet1.zip › Supplementary material/Fig S3.tif]

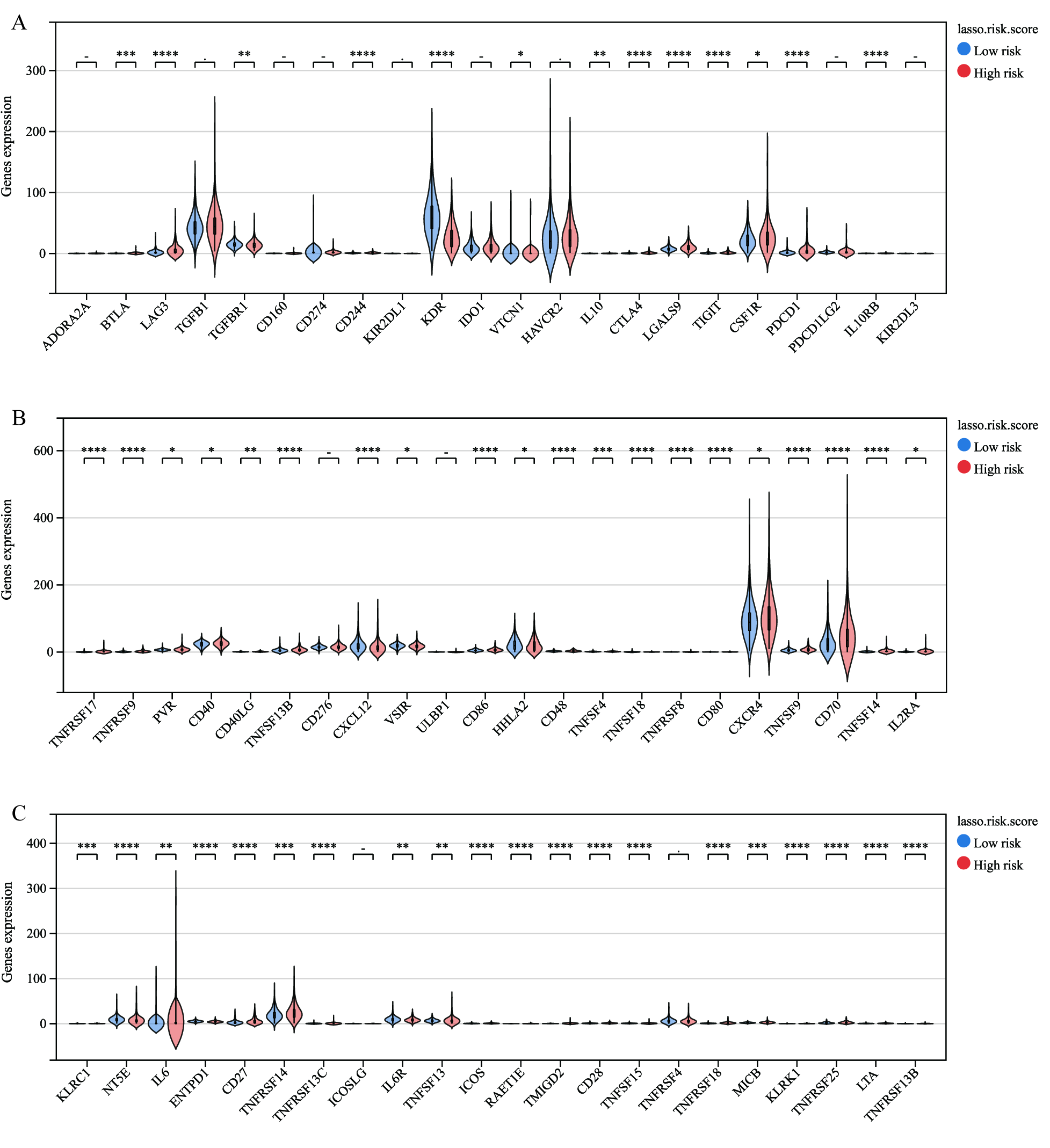

Supplement: Supplementary Figure 1 — The screening process of the PAQR6 gene. [file DataSheet1.zip › Supplementary material/Fig S4.tif]

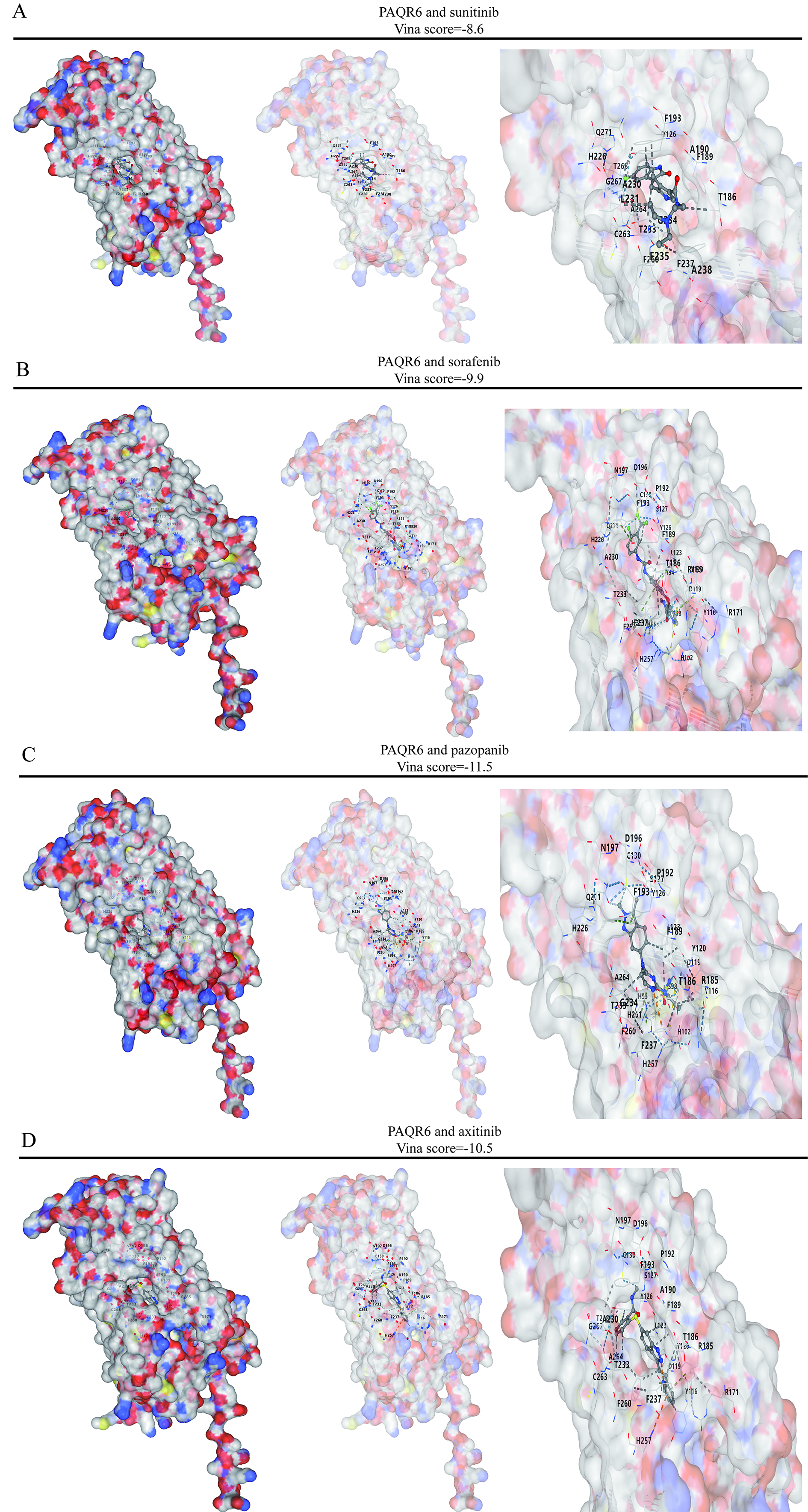

Supplement: Supplementary Figure 1 — The screening process of the PAQR6 gene. [file DataSheet1.zip › Supplementary material/Fig S5.tif]

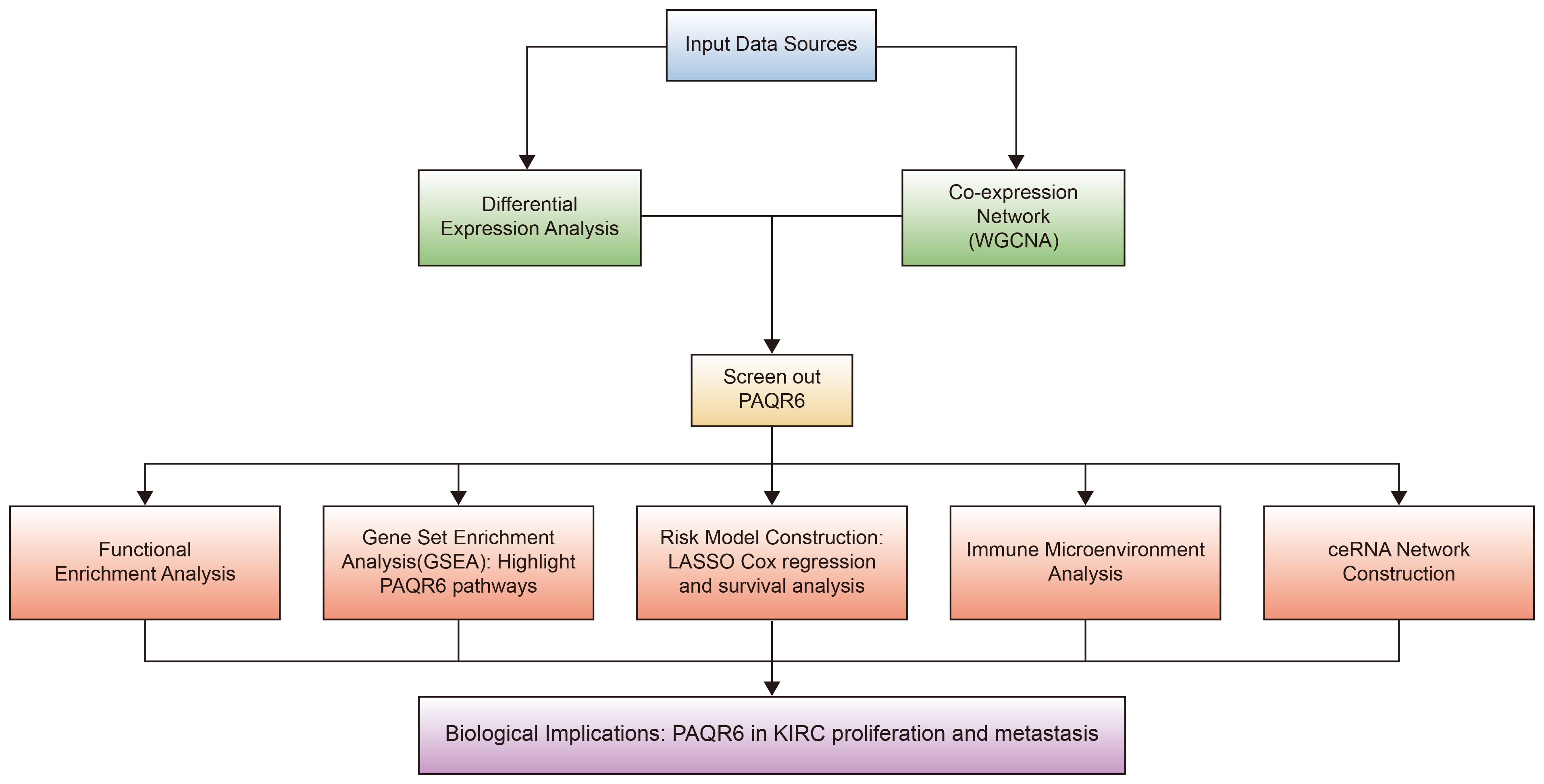

Supplement: Supplementary Figure 1 — The screening process of the PAQR6 gene. [file DataSheet1.zip › Supplementary material/Flowchart.tif]
